# Supplementary material for: A spectral method for assessing and combining multiple data visualizations
Source: Nat Commun. 2023 Feb 11;14:780. doi: 10.1038/s41467-023-36492-2 (PMC9922271; doi:10.1038/s41467-023-36492-2)
Supplement: Supplementary file 1 — Supplementary Information [file 41467_2023_36492_MOESM1_ESM.pdf]

Supplement to "A Spectral Method for Assessing and Combining Multiple Data Visualizations"

Rong Ma[1], Eric D. Sun[2] and James Zou[2]

Department of Statistics, Stanford University[1]
Department of Biomedical Data Science, Stanford University[2]

Contents

A Supplementary Results and Figures from Real Data Analysis 1
A.1 Silhouette Index 1
A.2 Additional Details of Implementation and Supplementary Figures 2

B Proof of Main Theorems 6
B.1 Notations 6
B.2 Sufficient Condition for (C1a) 6
B.3 Eigenscore Consistency: Proof of Theorem 1 12
B.4 Guarantee of Meta-Visualization: Proof of Theorem 2 16
B.5 Robustness of Spectral Weighting: Proof of Theorem 3 19
B.6 Necessity of the Signal Strength Condition (C2) 21
B.7 Proof of Auxiliary Lemma 1 22

A Supplementary Results and Figures from Real Data Analysis

A.1 Silhouette Index

Consider a partition $\{1,2,...,n\} = C_1 \cup C_2 \cup ... \cup C_K$, of n samples into K non-overlapping subsets, with each cluster containing at least 2 samples, being the true cluster membership of n samples. Let $d(i,j)$ be the distance between samples i and j in certain vector space. For each sample $i \in C_k$ for some $k \in \{1,2,...,K\}$, we define

$$a(i) = \frac{1}{|C_k|-1}\sum_{j\in C_k\setminus\{i\}} d(i,j), \tag{A.1}$$

as the mean distance between sample i and all other samples in cluster C_k, and define

$$b(i) = \min_{k'\neq k}\frac{1}{|C_{k'}|}\sum_{j\in C_{k'}} d(i,j), \tag{A.2}$$

as the smallest mean distance of i to all samples in any other cluster, of which sample i is not a member. Then the Silhouette index of sample i is defined as

$$SI(i) = \frac{b(i)-a(i)}{\max\{a(i),b(i)\}}. \tag{A.3}$$

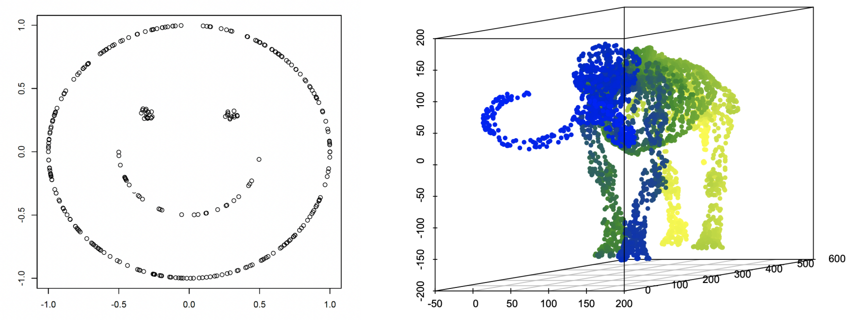

Figure 1: Low-dimensional structures used for simulations. Left: data points uniformly sampled from the two-dimensional "smiley face." Right: data points uniformly sampled from the three-dimensional "mammoth" manifold.

By definition, we have $SI(i) \in (-1, 1)$, and a higher $SI(i)$ indicates better concordance between the distances $\{d(i,j)\}_{j \neq i}$ and the underlying true cluster membership.

A.2 Additional Details of Implementation and Supplementary Figures

For the 16 candidate visualizations, we use the R functions below and recommend the following settings of tuning parameters:

- PCA: the fast SVD function `svds` from R package `rARPACK` with embedding dimension `k=2`.
- MDS: the basic R function `cmdscale` with embedding dimension `k=2`.
- Sammon: the R function `sammon` from R package `MASS` with embedding dimension `k=2`.
- LLE: the R function `lle` from R package `lle` with parameters `m=2, k=20, reg=2`.
- HLLE: the R function `embed` from R package `dimRed` with parameters `method="HLLE", knn=20, ndim=2`.
- Isomap: the R function `embed` from R package `dimRed` with parameters `method="Isomap", knn=20, ndim=2`.
- kPCA1&2: the R function `embed` from R package `dimRed` with parameters `method="kPCA", kpar=list(sigma=width), ndim=2`, where we set `width=0.01` for kPCA1 and `width=0.001` for kPCA2.
- LEIM: the R function `embed` from R package `dimRed` with parameters `ndim=2` and `method = "LaplacianEigenmaps"`.
- UMAP1&2: the R function `umap` from R package `uwot` with parameters `n_neighbors=n, n_components=2`, where we set `n=30` for UMAP1 and `width=50` for UMAP2.
- tSNE1&2: the R function `embed` from R package `dimRed` with parameters `method="tSNE", perplexity=n, ndim=2`, where we set `n=10` for tSNE1 and `n=50` for tSNE2.
- PHATE1&2: the R function `phate` from R package `phateR` with parameters `knn=n, ndim=2`, where we set `n=30` for PHATE1 and `n=50` for PHATE2.

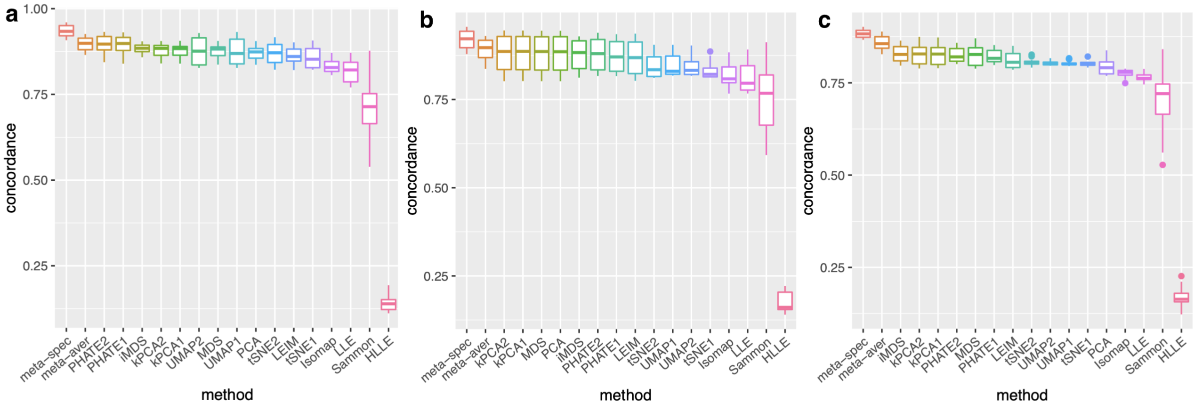

Figure 2: Concordance comparison from simulation studies. Boxplots (center line, median; box limits, upper and lower quartiles; points, outliers) for the mean concordance over n samples under each simulation setting for the 16 candidate visualizations and the 2 meta-visualizations, with each boxplot containing 20 mean concordances associated with 20 equispaced values of θ. (a) $n = 900$ independent samples generated from the Gaussian mixture model; (b) $n = 500$ independent samples generated from the smiley face model; (c) $n = 500$ independent samples generated from the mammoth model. The proposed spectral meta-distance matrix had superior performance than the others.

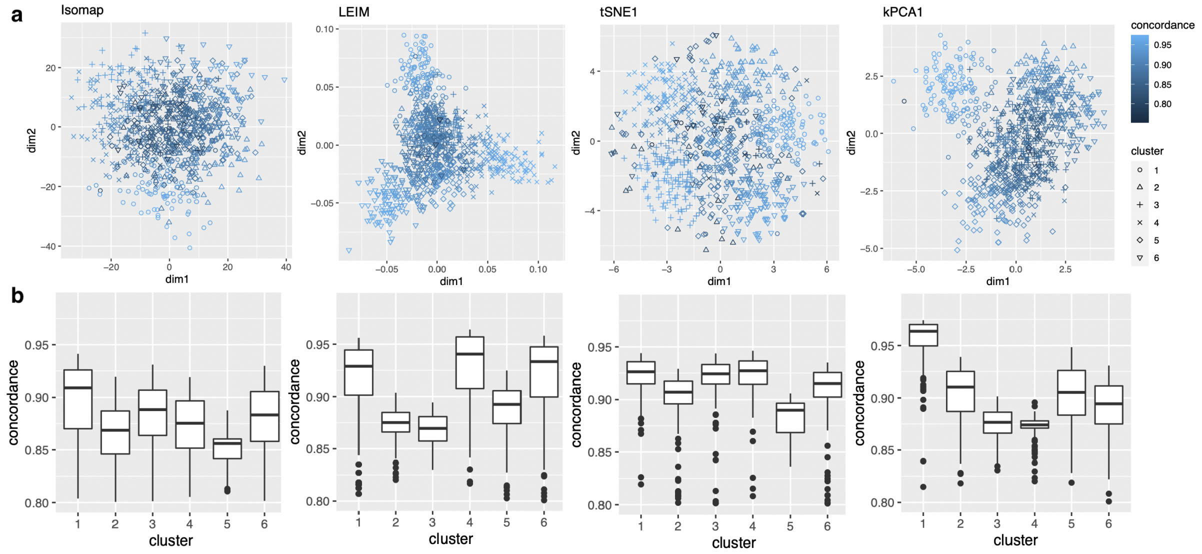

Figure 3: Additional visualizations of simulated Gaussian mixture data. Examples of candidate visualizations of the simulated data generated from $n = 900$ samples of a 6-class Gaussian mixture model, along with their pointwise concordance (a), and boxplots (center line, median; box limits, upper and lower quartiles; points, outliers) of $n = 900$ samples grouped by clusters (b). The plots indicates strengths and weaknesses of different methods in capturing the underlying clusters.

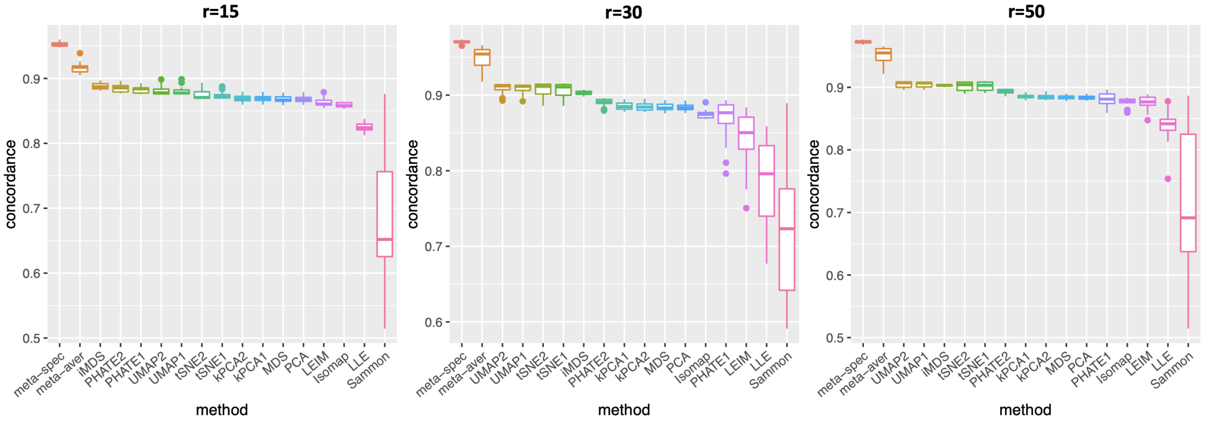

Figure 4: Concordance of simulated Gaussian mixture data with different intrinsic dimensions. Boxplots (center line, median; box limits, upper and lower quartiles; points, outliers) for the mean concordances over $n = 900$ samples under the Gaussian mixture model (i.e., setting (i)) for the 16 candidate visualizations and the 2 meta-visualizations, under various intrinsic dimensions (r), with each boxplot containing 20 mean concordances associated with 20 different values of $\theta \in [5, 10]$. The plots demonstrate the flexibility of the proposed method with respect to the intrinsic dimension r.

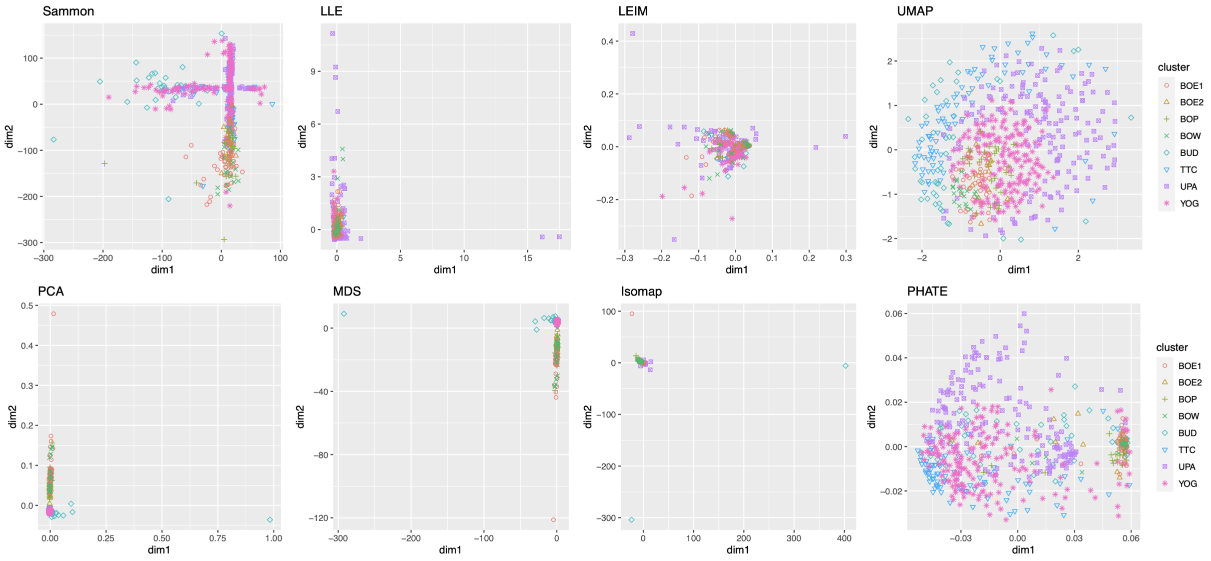

Figure 5: More examples of visualization of 590 fragments of religious text. The cluster patterns are far from clear based on individual methods. Source data are provided as a Source Data file.

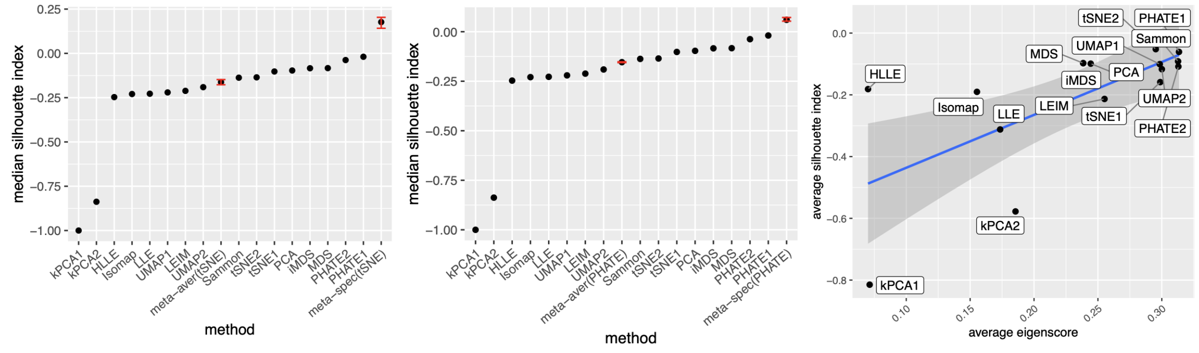

Figure 6: Silhouette indices from 590 fragments of religious text. Left: median Silhouette indices over $n = 590$ samples for 16 candidate and 2 meta-visualizations and the original data (ori), where the two meta-visualizations are based on t-SNE. The error bars indicate 95% confidence interval over 50 rounds of repetitions. Middle: median Silhouette indices for 16 candidate and 2 meta-visualization and the original data, where the two meta-visualizations are based on PHATE. The error bars indicate 95% confidence interval over 50 rounds of repetitions. Right: scatter plot of averaged eigenscores and averaged Silhouette indices over $n = 590$ samples for 16 candidate visualizations. The blue line and shaded area represent the linear regression fit of the 16 data points and their 95% predictive intervals. The left and middle panels indicate the improvement on performance was not sensitive to specific visualization methods. The right panel shows the potential interpretation and the effectiveness of the eigenscores. Source data are provided as a Source Data file.

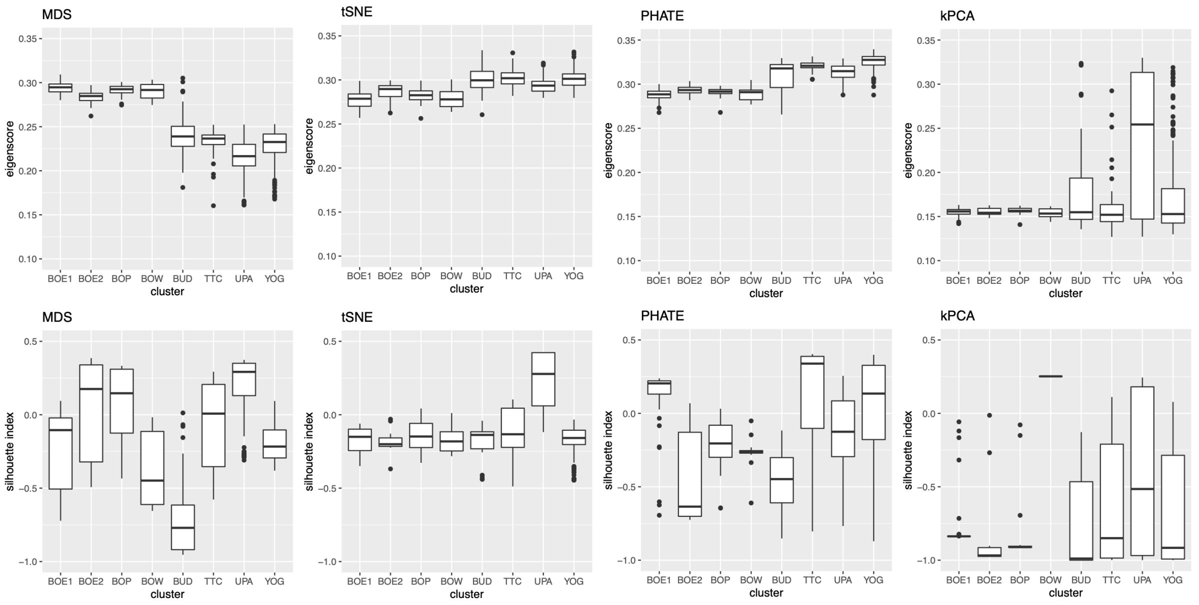

Figure 7: Eigenscores and silhouette indices for the religious text data. Boxplots (center line, median; box limits, upper and lower quartiles; points, outliers) of eigenscores (top) and Silhouette indices (bottom) over $n = 590$ samples for four candidate visualizations, grouped by clusters. Notable correlation between the two quantities indicates the effectiveness of the spectral weighting approach. Source data are provided as a Source Data file.

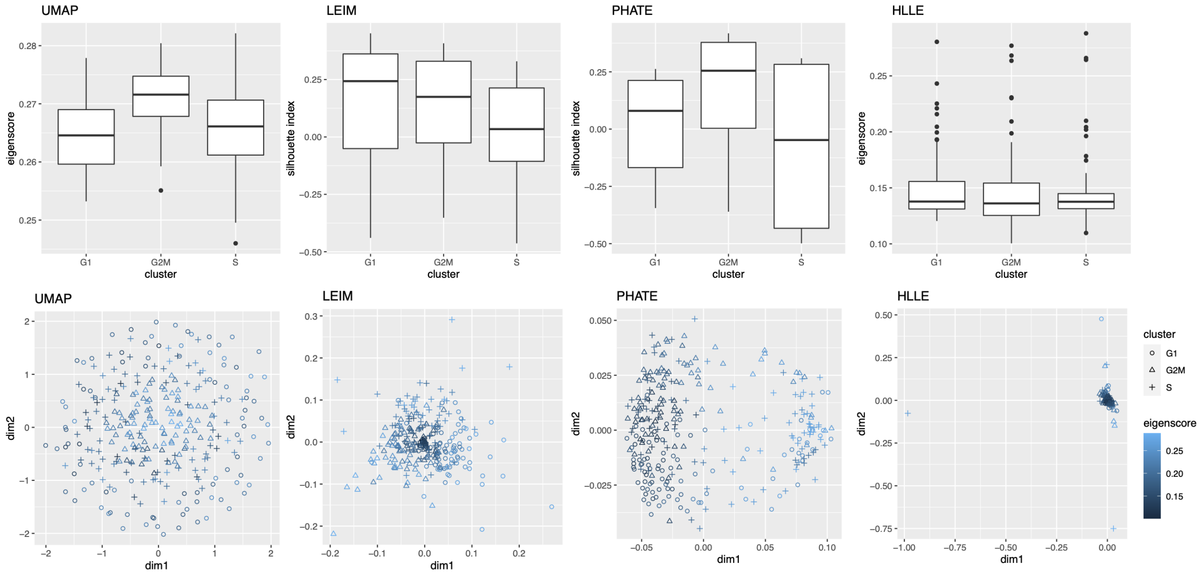

Figure 8: Illustrations of eigenscores for four candidate visualizations of the cell cycle data. The first row contains boxplots (center line, median; box limits, upper and lower quartiles; points, outliers) of eigenscores over $n = 288$ samples of the cell cycle data, grouped by the three cell cycle stages. Different visualizations may contribute to the meta-visualization from distinct aspects. Lighter color indicates better concordance to the underlying structures as assessed by the eigenscores. Source data are provided as a Source Data file.

B Proof of Main Theorems

B.1 Notations

For a vector $\mathbf{a} = (a_1, ..., a_n)^\top \in \mathbb{R}^n$, we denote $\text{diag}(a_1, ..., a_n) \in \mathbb{R}^{n\times n}$ as the diagonal matrix whose i-th diagonal entry is a_i, and define the ℓ_p norm $\|\mathbf{a}\|_p = \left(\sum_{i=1}^n a_i^p\right)^{1/p}$ and the ℓ_∞ norm $\|\mathbf{a}\|_\infty = \max_{1\le i\le n} |a_i|$. For a matrix $\mathbf{A} = (a_{ij}) \in \mathbb{R}^{n\times n}$, we define its Frobenius norm as $\|\mathbf{A}\|_F = \sqrt{\sum_{i=1}^n \sum_{j=1}^n a_{ij}^2}$, and its spectral norm as $\|\mathbf{A}\| = \sup_{\|\mathbf{x}\|_2\le 1} \|\mathbf{A}\mathbf{x}\|_2$; we also denote $\mathbf{A}_{.i} \in \mathbb{R}^n$ as its i-th column and $\mathbf{A}_{i.} \in \mathbb{R}^n$ as its i-th row. For sequences $\{a_n\}$ and $\{b_n\}$, we write $a_n = o(b_n)$ or $b_n \gg a_n$ if $\lim_n a_n/b_n = 0$, and write $a_n = O(b_n)$, $a_n \lesssim b_n$ or $b_n \gtrsim a_n$ if there exists a constant C such that $a_n \le C b_n$ for all n. We write $a_n \asymp b_n$ if $a_n \lesssim b_n$ and $a_n \gtrsim b_n$.

B.2 Sufficient Condition for (C1a)

We provide a sufficient condition in light of the signal-plus-noise model (2) of the main text with some intuitions that implies the sub-Gaussian condition (C1a) for $\mathbf{h}_i^{(k)}$ in (3). In general, the distortion vector $\mathbf{h}_i^{(k)}$ is jointly determined by the noise $\{\mathbf{Z}_i\}_{1\le i\le n}$, the noiseless samples $\{\mathbf{Y}_i^*\}_{1\le i\le n}$ and the dimension reduction map $f_k : \mathbb{R}^p \to \mathbb{R}^2$ associated to the k-th visualization method. Accordingly, our sufficient condition for (C1a) essentially involves regularity of the signal structure $\{\mathbf{Y}_i^*\}_{1\le i\le n}$ and the dimension reduction (DR) maps $f_k : \mathbb{R}^p \to \mathbb{R}^2$ for $1 \le k \le K$, and sub-Gaussianity of the noise vector $\{\mathbf{Z}_i\}_{1\le i\le n}$. We first state precisely our sufficient condition.

(C01) (*Regularity of the signal and DR map*) The noiseless samples $\{\mathbf{Y}_i^*\}_{1\le i\le n}$ lie on a bounded manifold $\mathcal{M}$ embedded in $\mathbb{R}^p$, and the DR map f_k and its first-order derivative are bounded

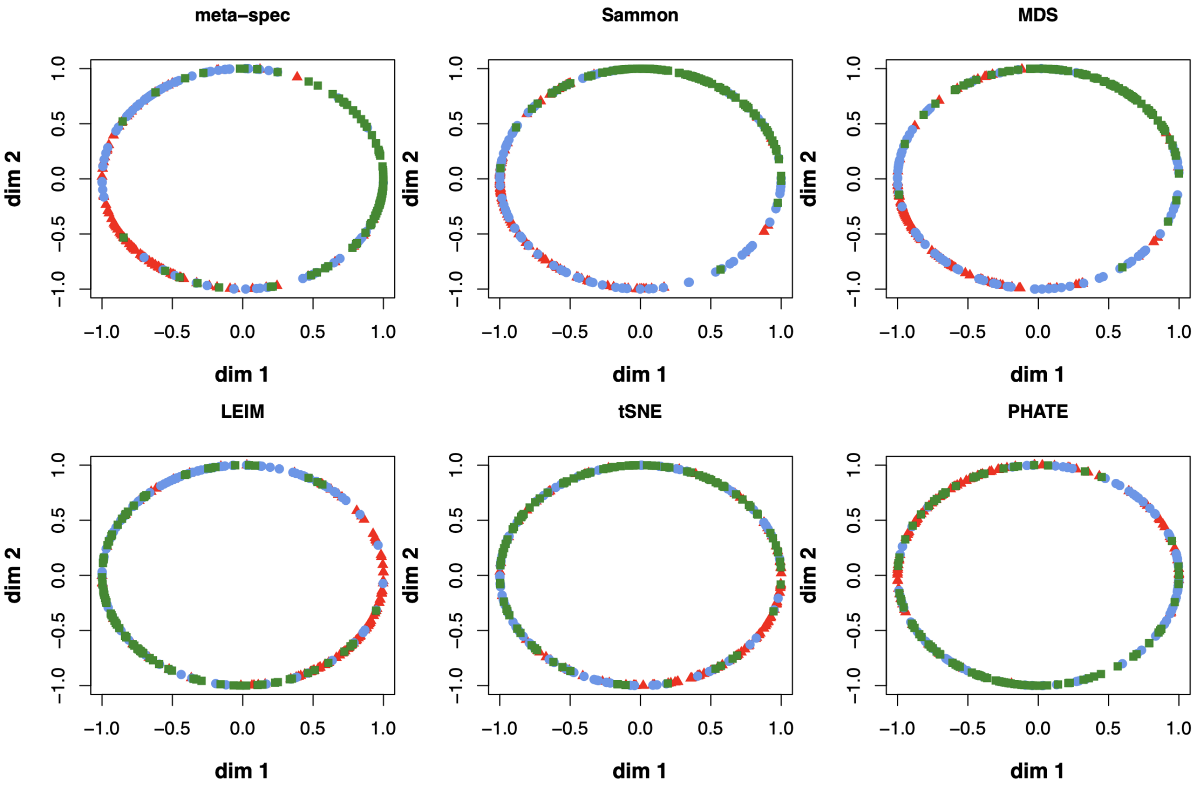

Figure 9: Examples of projection of candidate visualization onto the unit circle centred at the origin, from which the Kendall's tau statistics were computed. The colors correspond to the three cell cycle stages, suggesting that the spectral meta-visualization recovered the cell cycle better than other methods. Source data are provided as a Source Data file.

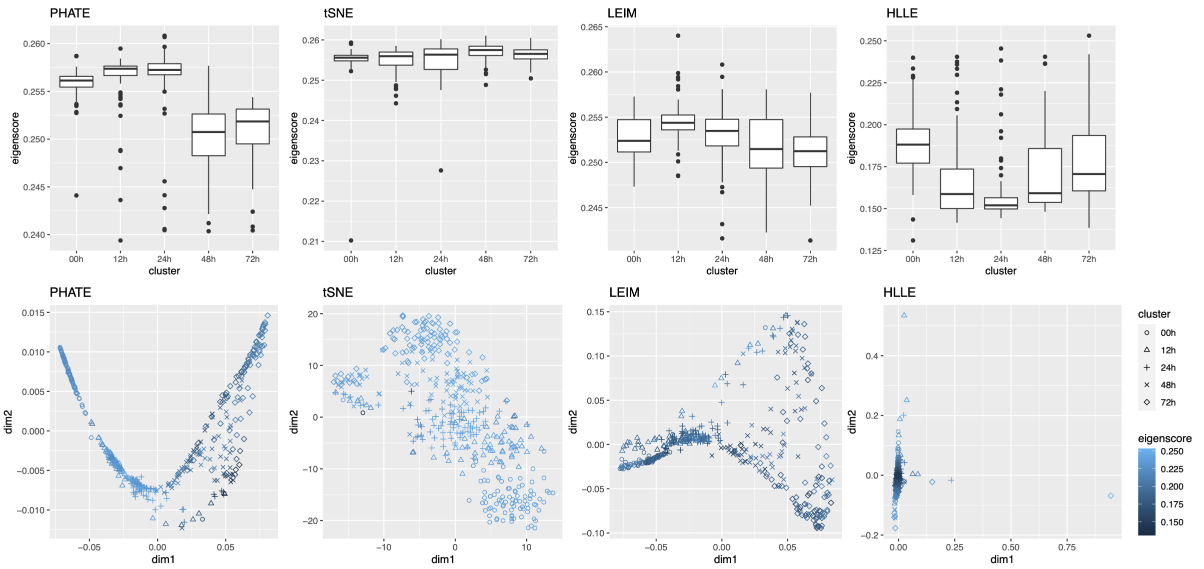

Figure 10: Illustrations of eigenscores for four candidate visualizations of the cell differentiation data. The first row contains boxplots (center line, median; box limits, upper and lower quartiles; points, outliers) of eigenscores over $n = 421$ samples of the cell differentiation data, grouped by the five time labels. Different visualizations may contribute to the meta-visualization from distinct aspects. Lighter color indicates better concordance to the underlying structures as assessed by the eigenscores. Source data are provided as a Source Data file.

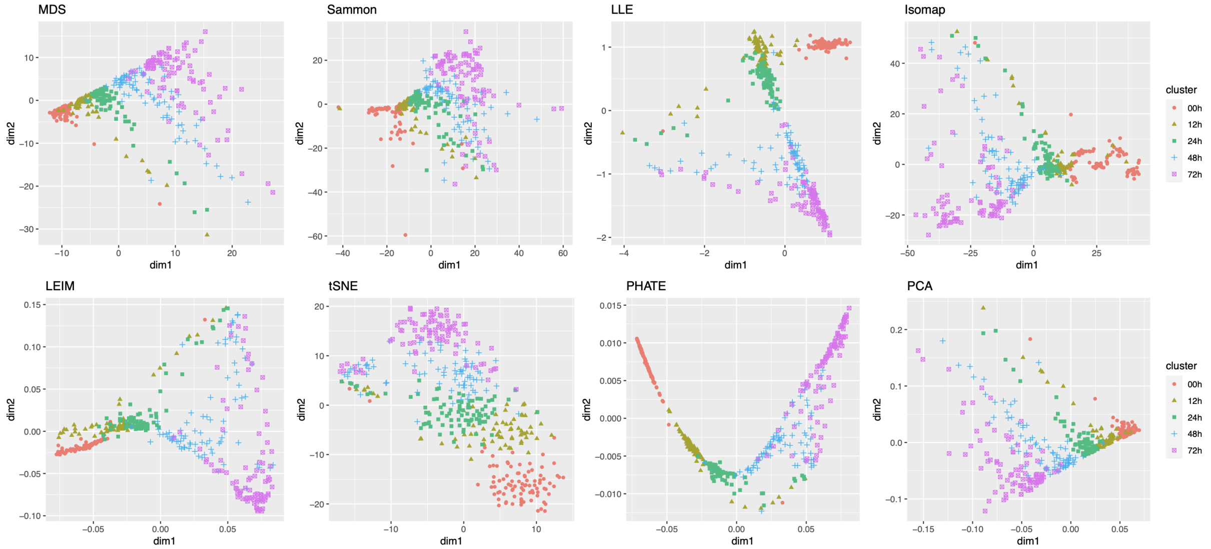

Figure 11: More examples of visualization of the 421 cells under differentiation. Source data are provided as a Source Data file.

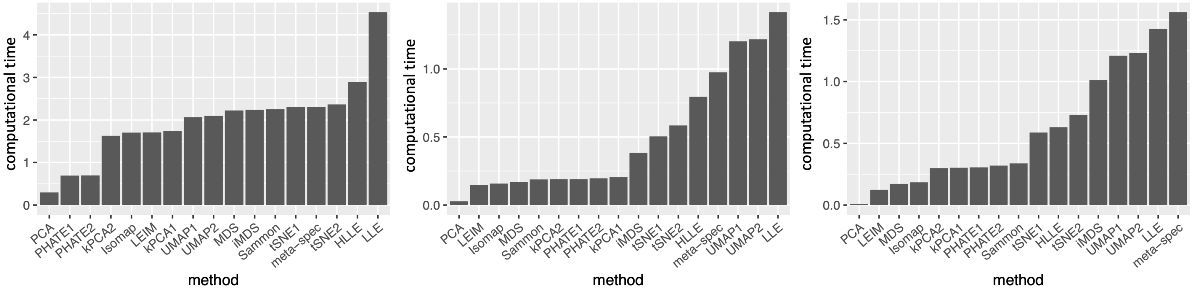

Figure 12: Computational time comparison for the data in the main text. Computational time (log of running time in seconds) for each candidate visualization and the proposed method, including all four steps of Algorithm 1, for the three real-world datasets considered in the main text. Left: religious and biblical text data; Middle: cell cycle data; Right: cell differentiation data. The proposed method had a computational time comparable to that of tSNE or UMAP for generating a single candidate visualization. Source data are provided as a Source Data file.

in the sense that for all $\mathbf{Y} \in \mathbb{R}^p$

$$L^{-1} \leq \|f_k(\mathbf{Y})\|_2 \leq L, \qquad \left\|\frac{\partial f_k(\mathbf{Y})}{\partial \mathbf{Y}}\right\| \leq C_f, \tag{B.1}$$

almost surely for some constants $L > 1$ and $C_f > 0$.

(C02) (*Sub-Gaussian noise*) The noise vectors $\{\mathbf{Z}_i\}_{1\leq i\leq n}$ are sub-Gaussian random vectors.

Intuitively, (C01) requires that the underlying signal structure is finite and that the DR map is also finite and sufficiently smooth. In particular, we allow that f_k is random in itself, as in the cases of randomized algorithms such as t-SNE and UMAP. The sub-Gaussian condition (C02) on the noise vector $\mathbf{Z}_i$ is mild and allows for wide range of noise structures.

Below we show that Conditions (C01) and (C02) jointly imply the sub-Gaussianity of $\mathbf{h}_i^{(k)}$. Firstly, note that by definition $\mathbf{P}_{i.}^{(k)} = (\|f_k(\mathbf{Y}_i) - f_k(\mathbf{Y}_1)\|_2, \|f_k(\mathbf{Y}_i) - f_k(\mathbf{Y}_2)\|_2, ..., \|f_k(\mathbf{Y}_i) - f_k(\mathbf{Y}_n)\|_2)^\top$. Then for each i, we can define the pairwise distance for the noiseless samples associated with the k-th visualization method as

$$\mathbf{P}_{i.}^{*(k)} = (\|f_k(\mathbf{Y}_i^*) - f_k(\mathbf{Y}_1^*)\|_2, \|f_k(\mathbf{Y}_i^*) - f_k(\mathbf{Y}_2^*)\|_2, ..., \|f_k(\mathbf{Y}_i^*) - f_k(\mathbf{Y}_n^*)\|_2)^\top. \tag{B.2}$$

Recall that $\mathbf{P}_{i.}^* = (\|\mathbf{Y}_i^* - \mathbf{Y}_1^*\|_2, \|\mathbf{Y}_i^* - \mathbf{Y}_2^*\|_2, ..., \|\mathbf{Y}_i^* - \mathbf{Y}_n^*\|_2)^\top$. Then, by (3), it follows that

$$\begin{aligned}\mathbf{h}_i^{(k)} &= c_k^{-1}\mathbf{P}_{i.}^{(k)} - \mathbf{P}_{i.}^* \\ &= (c_k^{-1}\mathbf{P}_{ij}^{*(k)} + \frac{c_k^{-1}\mathbf{g}_{k,ij}^\top[f_k(\mathbf{Y}_i) - f_k(\mathbf{Y}_j) - f_k(\mathbf{Y}_i^*) - f_k(\mathbf{Y}_j^*)]}{\|\mathbf{g}_{k,ij}\|_2} - \mathbf{P}_{ij}^*)_{1\leq j\leq n} \qquad \text{(B.3)} \\ &= \left(c_{i,k}^{-1}\mathbf{P}_{ij}^{*(k)} - \mathbf{P}_{ij}^* + \frac{c_{i,k}^{-1}\mathbf{g}_{k,ij}^\top}{\|\mathbf{g}_{k,ij}\|_2}\left[\frac{\partial f_k(\mathbf{Y})}{\partial \mathbf{Y}}\bigg|_{\mathbf{Y}=\mathbf{s}_i}\mathbf{Z}_i - \frac{\partial f_k(\mathbf{Y})}{\partial \mathbf{Y}}\bigg|_{\mathbf{Y}=\mathbf{s}_j}\mathbf{Z}_j\right]\right)_{1\leq j\leq n}, \qquad \text{(B.4)}\end{aligned}$$

where in (B.3) we used Taylor expansion of $\mathbf{P}_{ij}^{(k)} = \|f_k(\mathbf{Y}_i) - f_k(\mathbf{Y}_j)\|_2$ at $f_k(\mathbf{Y}_i^*) - f_k(\mathbf{Y}_j^*)$ with $\mathbf{g}_{k,ij}$ being some point between $\mathbf{P}_{ij}^{(k)}$ and $\mathbf{P}_{ij}^{*(k)}$, and in (B.4) we used Taylor expansion of $f_k(\mathbf{Y}_i)$ at $\mathbf{Y}_i^*$ with $\mathbf{s}_i$ being some point between $\mathbf{Y}_i$ and $\mathbf{Y}_i^*$.

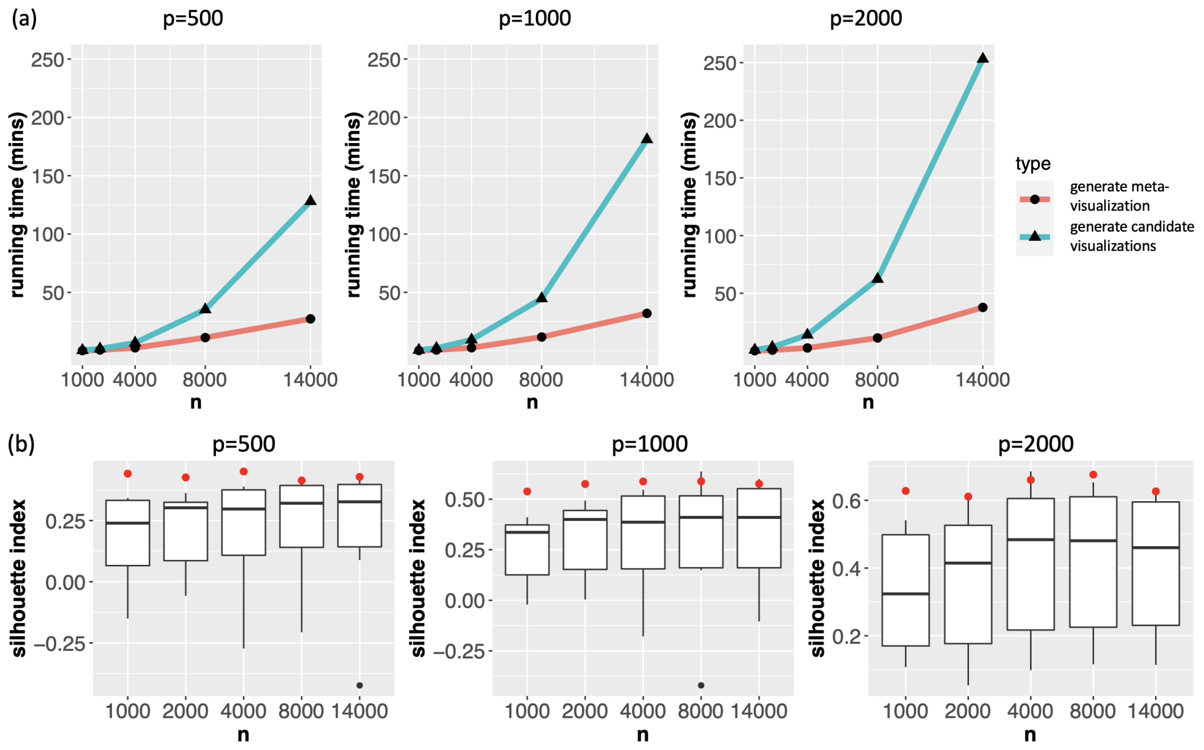

Figure 13: Computational time comparison for single-cell data of various sizes and dimensions. (a) Computational time (in mins) for generating 11 candidate visualizations ("generate candidate visualizations") for single-cell transcriptomic datasets of various sample sizes and dimensions, and that for generating the meta-visualizations ("generate meta-visualization") based on Algorithm 1. (b) Boxplots (center line, median; box limits, upper and lower quartiles; points, outliers) of the median Silhouette indices over $n = 1000, 2000, 4000, 8000$ and 14000 samples of the the single-cell transcriptomic data for 11 candidate visualizations and the meta-visualization (highlighted in red) with respect to the underlying true cell types. The running time of the proposed algorithm increased with n, but remained less than that for generating the candidate visualizations. For each n, when p increased, the running time for generating the candidate visualizations was longer, but the time cost for meta-visualization remained the same.

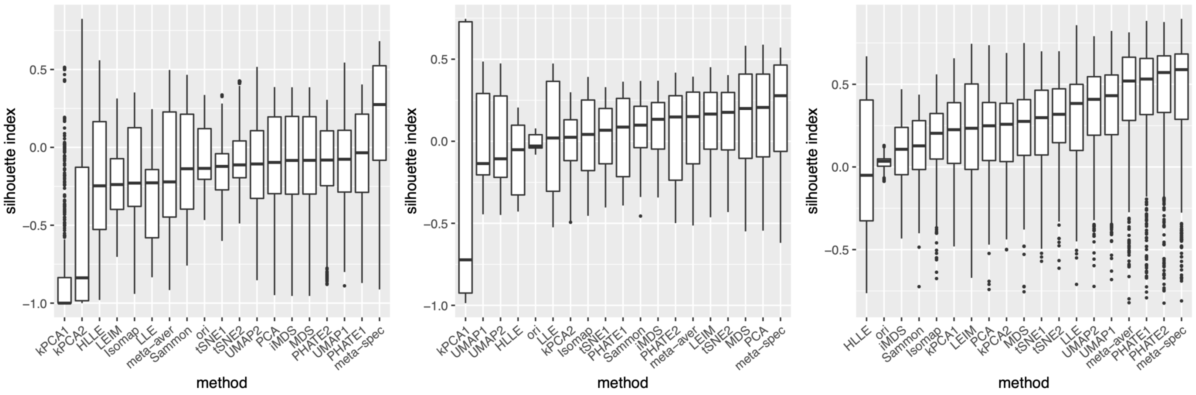

Figure 14: Limitations of original data. Boxplots (center line, median; box limits, upper and lower quartiles; points, outliers) of Silhouette index for 16 candidate, 2 meta-visualizations, and the original data (ori), over $n = 590$ (left panel), $n = 288$ (middle panel), and $n = 421$ (right panel) samples in the three datasets analyzed in the main paper. The original datasets showed significantly weaker cluster structure compared to most of the 16 candidate visualizations, suggesting that directly comparing a visualization with the noisy high-dimensional data may be misleading. Source data are provided as a Source Data file.

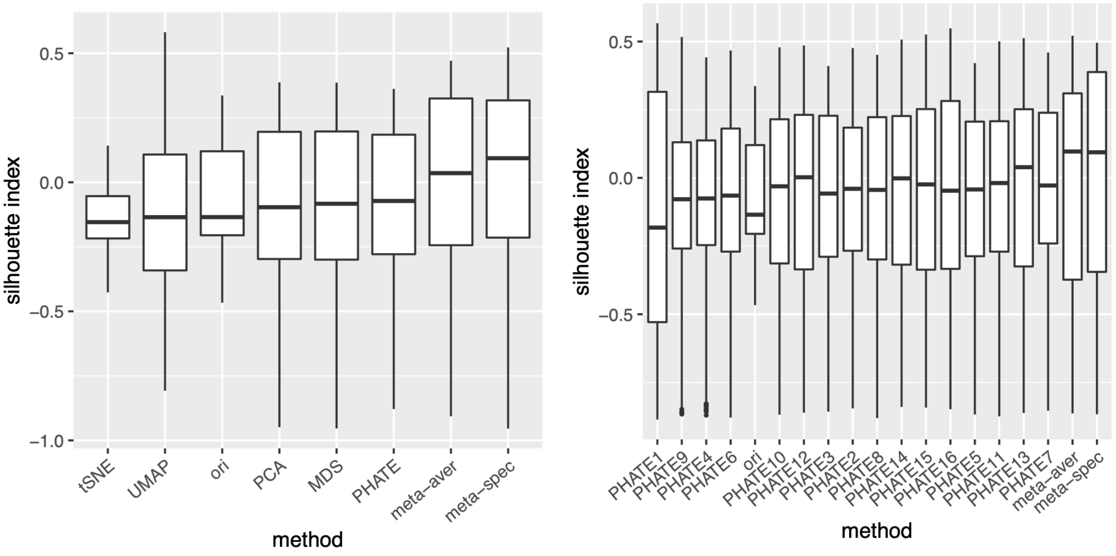

Figure 15: Additional Silhouette indices from the religious text data. Left: boxplots (center line, median; box limits, upper and lower quartiles; points, outliers) of Silhouette indices over $n = 590$ samples for 5 candidate and 2 meta-visualizations and the original data (ori), where the 5 candidate visualizations are based on t-SNE, PHATE, UMAP, PCA and MDS, respectively. Right: boxplots (center line, median; box limits, upper and lower quartiles; points, outliers) of Silhouette indices over $n = 590$ samples for 16 candidate and 2 meta-visualizations and the original data (ori), where the 16 candidate visualizations are based on PHATE with varying nearest neighbor parameters. Compared with Figure 3(d), they showed that the proposed method may benefit from additional, and more diverse candidate visualizations. Source data are provided as a Source Data file.

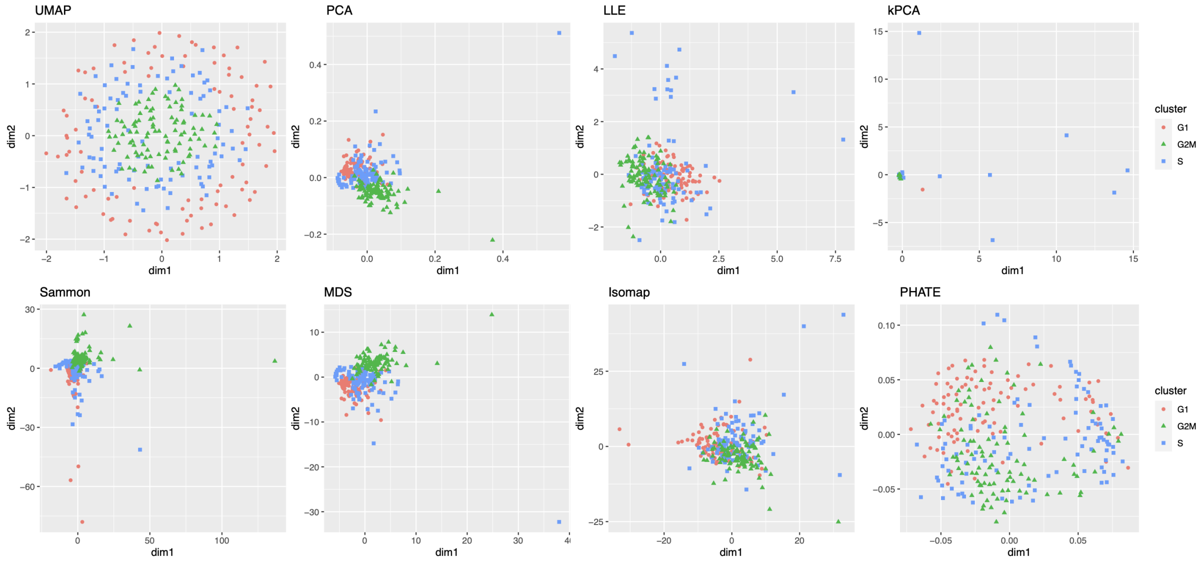

Figure 16: More examples of visualization of 288 mouse emryonic stem cells in cell cycles. Source data are provided as a Source Data file.

For each i, since $c_{i,k}$ is a parameter that accounts for the possible scaling difference caused by the dimension reduction map f_k, without loss of generality, we can take $c_{i,k} = \frac{\|f_k(\mathbf{Y}_i^*)\|_2}{\|\mathbf{Y}_i^*\|_2}$. Under Condition (C01), it follows that $c_k^{-1}\mathbf{P}_{ij}^{*(k)} - \mathbf{P}_{ij}^*$ in (B.4) is bounded and therefore a sub-Gaussian random variable. Similarly, for the random variable

$$\frac{c_{i,k}^{-1}\mathbf{g}_{k,ij}^\top}{\|\mathbf{g}_{k,ij}\|_2}\left[\frac{\partial f_k(\mathbf{Y})}{\partial \mathbf{Y}}\bigg|_{\mathbf{Y}=\mathbf{s}_i}\mathbf{Z}_i - \frac{\partial f_k(\mathbf{Y})}{\partial \mathbf{Y}}\bigg|_{\mathbf{Y}=\mathbf{s}_j}\mathbf{Z}_j\right] \tag{B.5}$$

in (B.4), under Condition (C01), we also have the boundedness of $\|\frac{\partial f_k(\mathbf{Y})}{\partial \mathbf{Y}}|_{\mathbf{Y}=\mathbf{s}_i}\|$ and $\|\frac{\partial f_k(\mathbf{Y})}{\partial \mathbf{Y}}|_{\mathbf{Y}=\mathbf{s}_j}\|$. By Proposition 2.5.2 of [2], these along with the boundedness of $c_{i,k}^{-1}$ and $\frac{\mathbf{g}_{k,ij}}{\|\mathbf{g}_{k,ij}\|_2}$, and the sub-Gaussianity of $\mathbf{Z}_i$ and $\mathbf{Z}_j$ from (C02), imply that (B.5) is also a sub-Gaussian random variable. Thus, we have verified that the sub-Gaussianity of $\mathbf{h}_i^{(k)}$ under Conditions (C01) and (C02). In particular, the sub-Gaussian parameter σ^2 in (C1a) is jointly determined by the underlying manifold $\mathcal{M}$, the scale (L) and the smoothness (C_f) of the DR map.

B.3 Eigenscore Consistency: Proof of Theorem 1

For simplicity, we omit the dependence on $i \in \{1, 2, ..., n\}$ and hereafter denote $\mathbf{x}_k = (x_{k1}, ..., x_{kn}) = \mathbf{P}_{i.}^{(k)}$, $\boldsymbol{y} = (y_1, ..., y_n) = \mathbf{P}_{i.}^*$, $\mathbf{z}_k = (z_{k1}, ..., z_{kn}) = \mathbf{h}_i^{(k)}$, $\widehat{\mathbf{s}} = \widehat{\mathbf{s}}_i$, $\mathbf{s} = \mathbf{s}_i$ and $c_k = c_{i,k}$. Therefore, model (3) in the main paper can be rewritten as

$$\mathbf{x}_k = c_k(\boldsymbol{y} + \mathbf{z}_k), \qquad k = 1, 2, ..., K. \tag{B.6}$$

We denote

$$\bar{\mathbf{x}}_k = \frac{\mathbf{x}_k}{\|\mathbf{x}_k\|_2} = \frac{\boldsymbol{y} + \mathbf{z}_k}{\|\boldsymbol{y} + \mathbf{z}_k\|_2}, \tag{B.7}$$

and define the matrix of normalized vectors

$$\bar{\mathbf{X}} = \begin{bmatrix} \bar{\mathbf{x}}_1^\top \\ \bar{\mathbf{x}}_2^\top \\ \vdots \\ \bar{\mathbf{x}}_K^\top \end{bmatrix} \in \mathbb{R}^{K \times n},$$

Then

$$\bar{\mathbf{X}}^\top \bar{\mathbf{X}} = (\bar{\mathbf{X}}_j^\top \bar{\mathbf{X}}_k)_{1 \le j,k \le n},$$

where $\bar{\mathbf{X}}_j \in \mathbb{R}^K$ is the j-th column of $\bar{\mathbf{X}}$, so that

$$\bar{\mathbf{X}}_j = \left(\frac{y_j + z_{1j}}{\|\boldsymbol{y} + \mathbf{z}_1\|_2}, \frac{y_j + z_{2j}}{\|\boldsymbol{y} + \mathbf{z}_2\|_2}, ..., \frac{y_j + z_{Kj}}{\|\boldsymbol{y} + \mathbf{z}_K\|_2} \right)^\top.$$

For any $1 \le j, k \le n$, it follows that

$$\begin{aligned}
\bar{\mathbf{X}}_j^\top \bar{\mathbf{X}}_k &= \sum_{i=1}^K \frac{(y_j + z_{ij})(y_k + z_{ik})}{\|\boldsymbol{y} + \mathbf{z}_i\|_2^2} \\
&= \left(\sum_{i=1}^K \|\boldsymbol{y} + \mathbf{z}_i\|_2^{-2} \right) y_j y_k + \left(\sum_{i=1}^K z_{ik} \|\boldsymbol{y} + \mathbf{z}_i\|_2^{-2} \right) y_j + \left(\sum_{i=1}^K z_{ij} \|\boldsymbol{y} + \mathbf{z}_i\|_2^{-2} \right) y_k + \sum_{i=1}^K z_{ij} z_{ik} \|\boldsymbol{y} + \mathbf{z}_i\|_2^{-2}.
\end{aligned}$$

This implies that

$$\bar{\mathbf{X}}^\top \bar{\mathbf{X}} = \|\mathbf{g}\|_2^2 \cdot \boldsymbol{y}\boldsymbol{y}^\top + \boldsymbol{y}\mathbf{g}^\top \mathbf{H}^\top + \mathbf{H}\mathbf{g}\boldsymbol{y}^\top + \mathbf{H}\mathbf{H}^\top, \tag{B.8}$$

where

$$\mathbf{g} = (\|\boldsymbol{y} + \mathbf{z}_1\|_2^{-1}, \|\boldsymbol{y} + \mathbf{z}_2\|_2^{-1}, ..., \|\boldsymbol{y} + \mathbf{z}_K\|_2^{-1}), \qquad \mathbf{H} = \begin{bmatrix} \frac{\mathbf{z}_1}{\|\boldsymbol{y}+\mathbf{z}_1\|_2} & \frac{\mathbf{z}_2}{\|\boldsymbol{y}+\mathbf{z}_2\|_2} & \cdots & \frac{\mathbf{z}_K}{\|\boldsymbol{y}+\mathbf{z}_K\|_2} \end{bmatrix} \in \mathbb{R}^{n \times K}$$

We denote

$$\bar{\mathbf{X}}^\top \bar{\mathbf{X}} = \|\mathbf{g}\|_2^2 \cdot \boldsymbol{y}\boldsymbol{y}^\top + \mathbf{E}, \qquad \mathbf{E} = \boldsymbol{y}\mathbf{g}^\top \mathbf{H}^\top + \mathbf{H}\mathbf{g}\boldsymbol{y}^\top + \mathbf{H}\mathbf{H}^\top, \tag{B.9}$$

Let the singular value decomposition of $\bar{\mathbf{X}}$ be

$$\bar{\mathbf{X}} = \sum_{i=1}^r \widehat{\sigma}_i \hat{\mathbf{u}}_i \hat{\mathbf{v}}_i^\top,$$

where $r = \min\{n, K\}$ and $\widehat{\sigma}_1 \ge \widehat{\sigma}_2 \ge ... \ge \widehat{\sigma}_r$. Then we have

$$\bar{\mathbf{X}}^\top \bar{\mathbf{X}} = \sum_{i=1}^r \widehat{\sigma}_i^2 \hat{\mathbf{v}}_i \hat{\mathbf{v}}_i^\top, \qquad \bar{\mathbf{X}}\bar{\mathbf{X}}^\top = \sum_{i=1}^r \widehat{\sigma}_i^2 \hat{\mathbf{u}}_i \hat{\mathbf{u}}_i^\top, \tag{B.10}$$

and

$$\mathbf{s} \cdot \|\boldsymbol{y}\|_2 = \left(\frac{\mathbf{x}_1^\top \boldsymbol{y}}{\|\mathbf{x}_1\|_2}, ..., \frac{\mathbf{x}_K^\top \boldsymbol{y}}{\|\mathbf{x}_K\|_2} \right)^\top = \bar{\mathbf{X}}\boldsymbol{y} = \sum_{i=1}^r \widehat{\sigma}_i \hat{\mathbf{u}}_i \hat{\mathbf{v}}_i^\top \boldsymbol{y}. \tag{B.11}$$

Therefore, we have

$$\begin{aligned}
1 - |\cos \angle(\hat{\mathbf{u}}_1, \mathbf{s})| &= 1 - |\cos \angle(\hat{\mathbf{u}}_1, \bar{\mathbf{X}}\boldsymbol{y})| = 1 - \frac{\widehat{\sigma}_1 |\hat{\mathbf{v}}_1^\top \boldsymbol{y}|}{\|\bar{\mathbf{X}}\boldsymbol{y}\|_2} \\
&= \frac{\|\bar{\mathbf{X}}\boldsymbol{y}\|_2 - \widehat{\sigma}_1 |\hat{\mathbf{v}}_1^\top \boldsymbol{y}|}{\|\bar{\mathbf{X}}\boldsymbol{y}\|_2} \le \frac{\| \sum_{i=2}^r \widehat{\sigma}_i \hat{\mathbf{u}}_i \hat{\mathbf{v}}_i^\top \boldsymbol{y}\|_2}{\|\bar{\mathbf{X}}\boldsymbol{y}\|_2} \le \frac{\widehat{\sigma}_2 \|\hat{\mathbf{V}}_{-1}^\top \boldsymbol{y}\|}{\widehat{\sigma}_1 |\hat{\mathbf{v}}_1^\top \boldsymbol{y}| - \widehat{\sigma}_2 \|\hat{\mathbf{V}}_{-1}^\top \boldsymbol{y}\|},
\end{aligned}$$

where $\widehat{\mathbf{V}}_{-1} = \begin{bmatrix}\hat{\mathbf{v}}_2 & ... & \hat{\mathbf{v}}_r\end{bmatrix} \in \mathbb{R}^{n\times(r-1)}$. Now we derive estimates of the random quantities $\widehat{\sigma}_1$, $\widehat{\sigma}_2$, $|\hat{\mathbf{v}}_1^\top \boldsymbol{y}|$ and $\|\widehat{\mathbf{V}}_{-1}^\top \boldsymbol{y}\|$ to obtain an upper bound of the last term in the above inequality. Consider the decomposition (B.9) and the first equation of (B.10). Firstly, by Weyl's inequality (e.g., Corollary III.2.6 of **(author?)** 3), we have

$$\left|\widehat{\sigma}_1^2 - \|\mathbf{g}\|_2^2 \cdot \|\boldsymbol{y}\|_2^2\right| \leq \|\mathbf{E}\|, \tag{B.12}$$

and

$$|\widehat{\sigma}_2^2| \leq \|\mathbf{E}\|. \tag{B.13}$$

Secondly, by Davis-Kahan's perturbation theorem (e.g., Theorem 1 of [4]), we have

$$\|\widehat{\mathbf{V}}_{-1}^\top \boldsymbol{y}\|/\|\boldsymbol{y}\| = \sqrt{1 - (\hat{\mathbf{v}}_1^\top \boldsymbol{y})^2/\|\boldsymbol{y}\|_2^2} \leq \frac{\|\mathbf{E}\|}{\|\mathbf{g}\|_2^2\|\boldsymbol{y}\|_2^2}. \tag{B.14}$$

Thus, if we denote $R = \|\mathbf{E}\|/(\|\mathbf{g}\|_2^2\|\boldsymbol{y}\|_2^2)$, by (B.12) and (B.13), if $R < 1$, we have

$$\frac{\widehat{\sigma}_2}{\widehat{\sigma}_1} \leq \frac{\|\mathbf{E}\|^{1/2}}{\sqrt{\|\mathbf{g}\|_2^2 \cdot \|\boldsymbol{y}\|_2^2 - \|\mathbf{E}\|}} = \frac{R^{1/2}}{\sqrt{1-R}},$$

and by (B.14), we have

$$\frac{\|\widehat{\mathbf{V}}_{-1}^\top \boldsymbol{y}\|}{|\hat{\mathbf{v}}_1^\top \boldsymbol{y}|} \leq \frac{R}{\sqrt{1-R^2}}.$$

Therefore, if $R < 1$, we have

$$1 - |\cos\angle(\hat{\mathbf{u}}_1, \mathbf{s})| \leq \frac{\frac{\widehat{\sigma}_2\|\widehat{\mathbf{V}}_{-1}^\top \boldsymbol{y}\|}{\widehat{\sigma}_1|\hat{\mathbf{v}}_1^\top \boldsymbol{y}|}}{1 - \frac{\widehat{\sigma}_2\|\widehat{\mathbf{V}}_{-1}^\top \boldsymbol{y}\|}{\widehat{\sigma}_1|\hat{\mathbf{v}}_1^\top \boldsymbol{y}|}} \leq \frac{\frac{R^{1/2}}{\sqrt{1-R}}\frac{R}{\sqrt{1-R^2}}}{1 - \frac{R^{1/2}}{\sqrt{1-R}}\frac{R}{\sqrt{1-R^2}}} = \frac{R^{3/2}}{\sqrt{(1-R)(1-R^2)} - R^{3/2}}. \tag{B.15}$$

If $R < 0.5$, we further have

$$1 - |\cos\angle(\hat{\mathbf{u}}_1, \mathbf{s})| \leq 4R^{3/2}. \tag{B.16}$$

The rest of the proof is devoted to the stochastic upper bound of R. Since by the elementary inequality $\|\mathbf{AB}\| \leq \|\mathbf{A}\| \cdot \|\mathbf{B}\|$ and triangle inequality, we have

$$\|\mathbf{E}\| \leq 2\|\boldsymbol{y}\|_2 \cdot \|\mathbf{g}\|_2 \cdot \|\mathbf{H}\| + \|\mathbf{H}\|^2. \tag{B.17}$$

Then it follows that

$$R = \frac{\|\mathbf{E}\|}{\|\mathbf{g}\|_2^2\|\boldsymbol{y}\|_2^2} \leq \frac{2\|\boldsymbol{y}\|_2\|\mathbf{g}\|_2\|\mathbf{H}\| + \|\mathbf{H}\|^2}{\|\mathbf{g}\|_2^2\|\boldsymbol{y}\|_2^2} = \frac{2\|\mathbf{H}\|}{\|\mathbf{g}\|_2\|\boldsymbol{y}\|_2} + \frac{\|\mathbf{H}\|^2}{\|\mathbf{g}\|_2^2\|\boldsymbol{y}\|_2^2}, \tag{B.18}$$

Hence, it suffices to obtain an upper bound for $\|\mathbf{H}\|$ and a lower bound for $\|\mathbf{g}\|_2$.

Upper bound of $\|\mathbf{H}\|$. Since

$$\mathbf{H} = \begin{bmatrix}\mathbf{z}_1 & \mathbf{z}_2 & ... & \mathbf{z}_K\end{bmatrix} \cdot \mathrm{diag}(\|\boldsymbol{y} + \mathbf{z}_1\|_2^{-1}, \|\boldsymbol{y} + \mathbf{z}_2\|_2^{-1}, ..., \|\boldsymbol{y} + \mathbf{z}_K\|_2^{-1}) \equiv \mathbf{H}_0 \cdot \mathrm{diag}(\mathbf{g}). \tag{B.19}$$

we have

$$\|\mathbf{H}\| \leq \|\mathbf{H}_0\| \cdot \|\mathbf{g}\|_\infty. \tag{B.20}$$

The following lemma gives the upper bound of $\|\mathbf{H}_0\|$, which is proved in Section B.7 below.

Lemma 1. *Under the conditions of Theorem 1, it holds that* $\|\mathbf{H}_0\| \leq C\sigma\sqrt{\rho}(\sqrt{n}+\sqrt{K})$ *with probability at least* $1-n^{-c}$ *for some universal constants* $C, c > 0$.

Now for

$$\|\mathbf{g}\|_\infty = \frac{1}{\min_{1\leq i\leq K}\|\boldsymbol{y}+\mathbf{z}_i\|_2},$$

note that

$$\|\boldsymbol{y}+\mathbf{z}_i\|_2^2 = \|\boldsymbol{y}\|_2^2 + \|\mathbf{z}_i\|_2^2 + 2\boldsymbol{y}^\top\mathbf{z}_i,$$

By Condition (C1a), we have

$$|\boldsymbol{y}^\top\mathbf{z}_i| \leq c'\sigma\|\boldsymbol{y}\|_2\sqrt{\log n} \qquad \text{in probability,}$$

for any small constant $c' > 0$, and that

$$\min_{1\leq i\leq K}\|\mathbf{z}_i\|_2^2 = cn\sigma^2(1+o(1)) \quad \text{in probability.}$$

Since $2\sigma\|\boldsymbol{y}\|_2\sqrt{\log n} \leq \sigma^2\log^2 n + \|\boldsymbol{y}\|_2^2/\log n$, we have

$$\min_{1\leq i\leq K}\|\boldsymbol{y}+\mathbf{z}_i\|_2^2 \geq \|\boldsymbol{y}\|_2^2 + cn\sigma^2(1+o(1)) - c'\sigma^2\log^2 n - c'\|\boldsymbol{y}\|_2^2/\log n \geq (1-\delta_n)\|\boldsymbol{y}\|_2^2 + (1-\delta_n)cn\sigma^2,$$

in probability, for some $\delta_n \to 0$.

In other words, we have

$$\min_{1\leq i\leq K}\|\boldsymbol{y}+\mathbf{z}_i\|_2 \geq (1-\delta_n)\|\boldsymbol{y}\|_2 + (1-\delta_n)c^{1/2}\sigma\sqrt{n}, \tag{B.21}$$

in probability, for some non-negative sequence $\delta_n \to 0$. This implies

$$\|\mathbf{H}\| \leq C\frac{\sigma\sqrt{\rho}(\sqrt{n}+\sqrt{K})}{\|\boldsymbol{y}\|_2 + \sigma\sqrt{n}}, \tag{B.22}$$

in probability.

Lower bound of $\|\mathbf{g}\|_2$. By similar argument that leads to (B.21), we have

$$\|\mathbf{g}\|_2^2 = \sum_{i=1}^K \|\boldsymbol{y}+\mathbf{z}_i\|_2^{-2} \geq K\min_{1\leq i\leq K}\|\boldsymbol{y}+\mathbf{z}_i\|_2^{-2} = \frac{K}{\max_{1\leq i\leq K}\|\boldsymbol{y}+\mathbf{z}_i\|_2^2} \geq \frac{K}{(1+\delta_n')[\|\boldsymbol{y}\|_2^2 + c\sigma^2 n]}, \tag{B.23}$$

in probability for some non-negative sequence $\delta_n' \to 0$.

Completing the proof. Combining the above results, we have

$$\frac{\|\mathbf{H}\|}{\|\mathbf{g}\|_2\|\boldsymbol{y}\|_2} \lesssim \frac{\sigma\sqrt{\rho}(\sqrt{n/K}+1)}{\|\boldsymbol{y}\|_2} \lesssim \frac{\sqrt{\rho n/K}}{\|\boldsymbol{y}\|/\sigma},$$

where the last inequality follows from $n > K$. Thus, under Condition (C2), for sufficiently large n, we always have $R < 0.5$, and therefore by (B.16), we have

$$\cos\angle(\hat{\mathbf{u}}_1, \mathbf{s}) \to 1, \qquad \text{in probability.} \tag{B.24}$$

Finally, note that for any $i = 1, 2, ..., K$, we have

$$s_i = \frac{\|\boldsymbol{y}\|_2^2 + \mathbf{z}_i^\top \boldsymbol{y}}{\|\boldsymbol{y} + \mathbf{z}_i\|_2 \cdot \|\boldsymbol{y}\|_2} \gtrsim \frac{\|\boldsymbol{y}\|_2^2 - c\sigma\|\boldsymbol{y}\|\sqrt{n/K}}{\|\boldsymbol{y}\|_2(\|\boldsymbol{y}\|_2 + \sigma\sqrt{n})} \geq \frac{\|\boldsymbol{y}\|_2 - c\sigma\sqrt{n/K}}{\|\boldsymbol{y}\|_2 + \sigma\sqrt{n}}, \tag{B.25}$$

in probability. By Condition (C2), whenever $K \ll n$ the right-hand side of the above inequality is strictly positive. In other words, in probability, $\mathbf{s}$ is a strictly positive vector. Thus, for $\widehat{\mathbf{s}} = |\hat{\mathbf{u}}_1|$, under the same event it holds that

$$\cos\angle(\widehat{\mathbf{s}}, \mathbf{s}) \leq \cos\angle(|\hat{\mathbf{u}}_1|, \mathbf{s}). \tag{B.26}$$

This along with (B.24) implies

$$\cos\angle(\widehat{\mathbf{s}}, \mathbf{s}) \to 1, \qquad \text{in probability.} \tag{B.27}$$

B.4 Guarantee of Meta-Visualization: Proof of Theorem 2

We still use the notation defined at the beginning of the proof of Theorem 1. In addition, we denote $\mathbf{x}^m = \bar{\mathbf{P}}^m_{i.}$, and define

$$\mathbf{x}^* = \sum_{i=1}^K w_i \bar{\mathbf{x}}_i, \tag{B.28}$$

where $w_i = s_i/\|\mathbf{s}\|_2$. To begin with, note that

$$\cos\angle(\mathbf{x}^*, \boldsymbol{y}) = \frac{\sum_{i=1}^K w_i \bar{\mathbf{x}}_i^\top \boldsymbol{y}}{\|\sum_{i=1}^K w_i \bar{\mathbf{x}}_i\|_2 \|\boldsymbol{y}\|_2},$$

$$\sum_{i=1}^K w_i \bar{\mathbf{x}}_i^\top \boldsymbol{y} = \frac{\sum_{i=1}^K (\bar{\mathbf{x}}_i^\top \boldsymbol{y})^2}{\sqrt{\sum_{i=1}^K (\bar{\mathbf{x}}_i^\top \boldsymbol{y})^2}} = \frac{\|\bar{\mathbf{X}}\boldsymbol{y}\|_2^2}{\sqrt{\sum_{i=1}^K (\bar{\mathbf{x}}_i^\top \boldsymbol{y})^2}} = \|\bar{\mathbf{X}}\boldsymbol{y}\|_2,$$

and

$$\left\|\sum_{i=1}^K w_i \bar{\mathbf{x}}_i\right\|_2 = \frac{\|\sum_{i=1}^K \bar{\mathbf{x}}_i^\top \boldsymbol{y} \bar{\mathbf{x}}_i\|_2}{\sqrt{\sum_{i=1}^K (\bar{\mathbf{x}}_i^\top \boldsymbol{y})^2}} = \frac{\|\bar{\mathbf{X}}^\top \bar{\mathbf{X}}\boldsymbol{y}\|_2}{\sqrt{\sum_{i=1}^K (\bar{\mathbf{x}}_i^\top \boldsymbol{y})^2}} = \frac{\|\bar{\mathbf{X}}^\top \bar{\mathbf{X}}\boldsymbol{y}\|_2}{\|\bar{\mathbf{X}}\boldsymbol{y}\|_2}.$$

Then

$$|\cos\angle(\mathbf{x}^*, \boldsymbol{y})| = \frac{\|\bar{\mathbf{X}}\boldsymbol{y}\|_2^2}{\|\bar{\mathbf{X}}^\top \bar{\mathbf{X}}\boldsymbol{y}\|_2 \|\boldsymbol{y}\|_2}.$$

Similarly, we have

$$\max_{1 \leq i \leq K} |\cos\angle(\mathbf{x}_i, \boldsymbol{y})| = \|\bar{\mathbf{X}}\boldsymbol{y}\|_\infty / \|\boldsymbol{y}\|_2.$$

We first prove some auxiliary results. Since

$$\bar{\mathbf{X}}^\top = \begin{bmatrix} \boldsymbol{y} + \mathbf{z}_1 & ...\boldsymbol{y} + \mathbf{z}_K \end{bmatrix} \cdot \text{diag}(\mathbf{g}),$$

we have, for sufficiently large n,

$$\begin{aligned} \|\bar{\mathbf{X}}\| &\leq \|\mathbf{g}\|_\infty \cdot \left\| \begin{bmatrix} \boldsymbol{y} + \mathbf{z}_1 & ...\boldsymbol{y} + \mathbf{z}_K \end{bmatrix} \right\| \leq \|\mathbf{g}\|_\infty \cdot (\sqrt{K}\|\boldsymbol{y}\|_2 + \|\mathbf{H}_0\|) \\ &\leq \|\mathbf{g}\|_\infty \cdot [\sqrt{K}\|\boldsymbol{y}\|_2 + C\sigma\sqrt{\rho}(\sqrt{n} + \sqrt{K})] \end{aligned} \tag{B.29}$$

in probability, according to Lemma 1, and

$$\|\bar{\mathbf{X}}\boldsymbol{y}\|_2 = \left\| \boldsymbol{y}^\top \begin{bmatrix} \boldsymbol{y} + \mathbf{z}_1 & ... \boldsymbol{y} + \mathbf{z}_K \end{bmatrix} \cdot \mathrm{diag}(\mathbf{g}) \right\|_2 \geq \min_{1 \leq i \leq K} |\|\boldsymbol{y}\|_2^2 + \boldsymbol{y}^\top \mathbf{z}_i| \cdot \|\mathbf{g}\|_2 \geq \|\mathbf{g}\|_2 \cdot (1 - \delta_n)\|\boldsymbol{y}\|_2^2, \tag{B.30}$$

$$\|\bar{\mathbf{X}}\boldsymbol{y}\|_\infty \leq \max_{1 \leq i \leq K} |\|\boldsymbol{y}\|_2^2 + \boldsymbol{y}^\top \mathbf{z}_i| \cdot \|\mathbf{g}\|_\infty \leq \|\mathbf{g}\|_\infty (1 + \delta_n)\|\boldsymbol{y}\|_2^2, \tag{B.31}$$

for some non-negative sequence $\delta_n \to 0$ as $n \to \infty$, in probability. In addition, by (B.23) and (B.21), we have

$$\|\mathbf{g}\|_2 \geq \frac{\sqrt{K}}{(1 + \delta_n)(\|\boldsymbol{y}\|_2 + c\sigma\sqrt{n})}, \qquad \|\mathbf{g}\|_\infty \leq \frac{1}{(1 - \delta_n)(\|\boldsymbol{y}\|_2 + c\sigma\sqrt{n})}. \tag{B.32}$$

in probability for some $\delta_n \to 0$. Now we show that

$$\cos \angle(\mathbf{x}^m, \mathbf{x}^*) \to 1. \tag{B.33}$$

To see this, since

$$\mathbf{x}^m = \sum_{i=1}^{K} \hat{s}_i \bar{\mathbf{x}}_i = \bar{\mathbf{X}}^\top \widehat{\mathbf{s}}, \tag{B.34}$$

and $\mathbf{x}^* = \bar{\mathbf{X}}^\top \mathbf{w}$ as in (B.28), we have

$$\cos \angle(\mathbf{x}^m, \mathbf{x}^*) \geq \frac{\mathbf{w}^\top \bar{\mathbf{X}}\bar{\mathbf{X}}^\top \widehat{\mathbf{s}}}{\|\bar{\mathbf{X}}^\top \widehat{\mathbf{s}}\|_2 \|\bar{\mathbf{X}}^\top \mathbf{w}\|_2} = \frac{\widehat{\mathbf{s}}^\top \bar{\mathbf{X}}\bar{\mathbf{X}}^\top \widehat{\mathbf{s}}}{\|\bar{\mathbf{X}}^\top \widehat{\mathbf{s}}\|_2 \|\bar{\mathbf{X}}^\top \mathbf{w}\|_2} + \frac{\widehat{\mathbf{s}}^\top \bar{\mathbf{X}}\bar{\mathbf{X}}^\top (\mathbf{w} - \widehat{\mathbf{s}})}{\|\bar{\mathbf{X}}^\top \widehat{\mathbf{s}}\|_2 \|\bar{\mathbf{X}}^\top \mathbf{w}\|_2}. \tag{B.35}$$

Hence

$$\begin{aligned}
|1 - \cos \angle(\mathbf{x}^m, \mathbf{x}^*)| &= \left| 1 - \frac{\|\bar{\mathbf{X}}^\top \widehat{\mathbf{s}}\|_2}{\|\bar{\mathbf{X}}^\top \mathbf{w}\|_2} + \frac{\widehat{\mathbf{s}}^\top \bar{\mathbf{X}}\bar{\mathbf{X}}^\top (\mathbf{w} - \widehat{\mathbf{s}})}{\|\bar{\mathbf{X}}^\top \widehat{\mathbf{s}}\|_2 \|\bar{\mathbf{X}}^\top \mathbf{w}\|_2} \right| \\
&\leq \frac{|\|\bar{\mathbf{X}}^\top \widehat{\mathbf{s}}\|_2 - \|\bar{\mathbf{X}}^\top \mathbf{w}\|_2|}{\|\bar{\mathbf{X}}^\top \mathbf{w}\|_2} + \frac{|\widehat{\mathbf{s}}^\top \bar{\mathbf{X}}\bar{\mathbf{X}}^\top (\widehat{\mathbf{s}} - \mathbf{w})|}{\|\bar{\mathbf{X}}^\top \widehat{\mathbf{s}}\|_2 \|\bar{\mathbf{X}}^\top \mathbf{w}\|_2} \\
&\leq \frac{2\|\bar{\mathbf{X}}\|\|\widehat{\mathbf{s}} - \mathbf{w}\|_2}{\|\bar{\mathbf{X}}^\top \mathbf{w}\|_2} \\
&\leq \frac{2\|\bar{\mathbf{X}}\|\|\bar{\mathbf{X}}\boldsymbol{y}\|_2 \|\widehat{\mathbf{s}} - \mathbf{w}\|_2}{\|\bar{\mathbf{X}}^\top \bar{\mathbf{X}}\boldsymbol{y}\|_2}.
\end{aligned}$$

Note that by Theorem 1, we have $\|\widehat{\mathbf{s}} - \mathbf{w}\|_2 \to 0$ in probability. Then it suffices to show that

$$\frac{\|\bar{\mathbf{X}}\|\|\bar{\mathbf{X}}\boldsymbol{y}\|_2}{\|\bar{\mathbf{X}}^\top \bar{\mathbf{X}}\boldsymbol{y}\|_2} = O_P(1). \tag{B.36}$$

To see this, since

$$\frac{\|\bar{\mathbf{X}}\|\|\bar{\mathbf{X}}\boldsymbol{y}\|_2}{\|\bar{\mathbf{X}}^\top \bar{\mathbf{X}}\boldsymbol{y}\|_2} \leq \frac{\|\bar{\mathbf{X}}\|\|\bar{\mathbf{X}}\boldsymbol{y}\|_2 \|\boldsymbol{y}\|_2}{|\boldsymbol{y}^\top \bar{\mathbf{X}}^\top \bar{\mathbf{X}}\boldsymbol{y}|} = \frac{\|\bar{\mathbf{X}}\|\|\boldsymbol{y}\|_2}{\|\bar{\mathbf{X}}\boldsymbol{y}\|_2}. \tag{B.37}$$

Note that

$$\bar{\mathbf{X}}^\top = (\begin{bmatrix} \boldsymbol{y} & ... & \boldsymbol{y} \end{bmatrix} + \mathbf{H}_0) \cdot \mathrm{diag}(\mathbf{g}). \tag{B.38}$$

By Condition (C2), whenever $K \ll n$, we have

$$\begin{aligned}
\boldsymbol{y}^\top \bar{\mathbf{X}}^\top &= (\begin{bmatrix} \|\boldsymbol{y}\|_2^2 & ... & \|\boldsymbol{y}\|_2^2 \end{bmatrix} + \boldsymbol{y}^\top \mathbf{H}_0) \cdot \mathrm{diag}(\mathbf{g}) \\
&= \begin{bmatrix} \|\boldsymbol{y}\|_2^2(1 + o(1)) & ... & \|\boldsymbol{y}\|_2^2(1 + o(1)) \end{bmatrix} \cdot \mathrm{diag}(\mathbf{g}),
\end{aligned}$$

in probability, which implies

$$\begin{aligned}\|\bar{\mathbf{X}}\boldsymbol{y}\|_2 &= \|\boldsymbol{y}\|_2^2(1+o(1))\sqrt{\sum_{i=1}^K \|\boldsymbol{y}+\mathbf{z}_i\|_2^{-2}} \\ &\asymp \|\boldsymbol{y}\|_2^2\sqrt{K}(\|\boldsymbol{y}\|_2+\sigma\sqrt{n})^{-1}\end{aligned}$$

in probability, where the last inequality follows from (B.21) and (B.23). On the one hand, we have

$$\begin{aligned}\|\bar{\mathbf{X}}\| &\le \| \begin{bmatrix}\boldsymbol{y} & \dots & \boldsymbol{y}\end{bmatrix} + \mathbf{H}_0\| \|\mathbf{g}\|_\infty \\ &\lesssim (\|\boldsymbol{y}\|_2+\sigma\sqrt{n})^{-1}(\sqrt{K}\|\boldsymbol{y}\|_2 + \sigma\rho(\sqrt{K}+\sqrt{n})) \\ &\lesssim \sqrt{K}\|\boldsymbol{y}\|_2(\|\boldsymbol{y}\|_2+\sigma\sqrt{n})^{-1}.\end{aligned}$$

Thus, we have

$$\frac{\|\bar{\mathbf{X}}\|\|\boldsymbol{y}\|_2}{\|\bar{\mathbf{X}}\boldsymbol{y}\|_2} = O_P(1), \tag{B.39}$$

which along with (B.37) implies (B.36). This completes our proof of (B.33). Finally, we note that

$$|\cos\angle(\mathbf{x}^*,\boldsymbol{y})| = \frac{\|\bar{\mathbf{X}}\boldsymbol{y}\|_2^2}{\|\bar{\mathbf{X}}^\top\bar{\mathbf{X}}\boldsymbol{y}\|_2\|\boldsymbol{y}\|_2} \ge \frac{\|\bar{\mathbf{X}}\boldsymbol{y}\|_2^2}{\|\bar{\mathbf{X}}\|\cdot\|\bar{\mathbf{X}}\boldsymbol{y}\|_2\|\boldsymbol{y}\|_2} = \frac{\|\bar{\mathbf{X}}\boldsymbol{y}\|_2}{\|\bar{\mathbf{X}}\|\cdot\|\boldsymbol{y}\|_2},$$

so that

$$1 - \frac{\|\bar{\mathbf{X}}\boldsymbol{y}\|_2}{\|\bar{\mathbf{X}}\|\|\boldsymbol{y}\|_2} \ge 1 - |\cos\angle(\mathbf{x}^*,\boldsymbol{y})|.$$

Now in order to prove the theorem, we show that, on the one hand,

$$1 - \frac{\|\bar{\mathbf{X}}\boldsymbol{y}\|_2}{\|\bar{\mathbf{X}}\|\|\boldsymbol{y}\|_2} \to 0 \qquad \text{in probability,} \tag{B.40}$$

and on the other hand, under the additional assumption that $\|\boldsymbol{y}\|_2/\sigma\sqrt{n} \le C$ for some sufficiently small constant,

$$\frac{\|\bar{\mathbf{X}}\boldsymbol{y}\|_\infty}{\|\boldsymbol{y}\|_2} \le 1-\delta, \qquad \text{in probability,} \tag{B.41}$$

for some constant $\delta > 0$.

To obtain (B.40), we note that by (B.29) (B.30) and (B.32), for some sequence $\delta_n \to 0$, we have

$$\begin{aligned}&\frac{\|\bar{\mathbf{X}}\|\|\boldsymbol{y}\|_2 - \|\bar{\mathbf{X}}\boldsymbol{y}\|_2}{\|\bar{\mathbf{X}}\|\|\boldsymbol{y}\|_2} = \frac{\|\boldsymbol{y}\|_2 - \|\bar{\mathbf{X}}\boldsymbol{y}\|_2/\|\bar{\mathbf{X}}\|}{\|\boldsymbol{y}\|_2} \le \frac{\|\boldsymbol{y}\|_2 - \frac{\|\mathbf{g}\|_2(1-\delta_n)}{\|\mathbf{g}\|_\infty(1+\delta_n)\sqrt{K}}}{\|\boldsymbol{y}\|_2} \\ &\le 1 - \frac{(1-\delta_n)^2(1+c\sigma\sqrt{n}/\|\boldsymbol{y}\|_2)}{(1+\delta_n)^2(1+c\sigma\sqrt{n}/\|\boldsymbol{y}\|_2)},\end{aligned}$$

in probability. To obtain (B.41), for some sequence $\delta_n \to 0$, we have

$$\frac{\|\bar{\mathbf{X}}\boldsymbol{y}\|_\infty}{\|\boldsymbol{y}\|_2} \le \|\mathbf{g}\|_\infty(1+\delta_n)\|\boldsymbol{y}\|_2 \le \frac{(1+\delta_n)}{(1-\delta_n)(1+c\sigma\sqrt{n}/\|\boldsymbol{y}\|_2)} \le 1-\delta, \tag{B.42}$$

in probability, as long as $\|\boldsymbol{y}\|_2/\sigma\sqrt{n} \le C$ for some sufficiently small constant $C > 0$. This completes our proof of Theorem 2.

B.5 Robustness of Spectral Weighting: Proof of Theorem 3

For the first statement concerning $\bar{\mathbf{P}}^m$, firstly, we note that by the proof of Theorem 2, we have $\cos\angle(\mathbf{x}^*, \boldsymbol{y}) \to 1$ under the conditions of Theorem 1, as the effects of the adversarial candidate visualizations vanish in (B.28). Therefore, it suffices to show that under such an adversarial setting, we still have $\cos\angle(\mathbf{x}^m, \mathbf{x}^*) \to 1$ in probability.

To see this, by the proof of Theorem 2, it suffices to show that $\|\widehat{\mathbf{s}} - \mathbf{w}\|_2 \to 0$ in probability, or,

$$\cos(\widehat{\mathbf{s}}, \mathbf{s}) \to 0, \qquad \text{in probability.} \tag{B.43}$$

By the proof of Theorem 1, we have

$$\bar{\mathbf{X}}_j = \left(\frac{y_j + z_{1j}}{\|\boldsymbol{y} + \mathbf{z}_1\|_2}, ..., \frac{y_j + z_{K'j}}{\|\boldsymbol{y} + \mathbf{z}'_K\|_2}, \frac{z_{K'+1,j}}{\|\mathbf{z}_{K'+1}\|_2}, ..., \frac{z_{Kj}}{\|\mathbf{z}_K\|_2} \right)^\top,$$

where we denote $\mathbf{z}_k = \mathbf{x}_k$ with $\mathbf{z}_k^\top \boldsymbol{y} = 0$ for all $k \geq K' + 1$. For any $1 \leq j, k \leq n$, it follows that

$$\begin{aligned}
\bar{\mathbf{X}}_j^\top \bar{\mathbf{X}}_k &= \sum_{i=1}^{K'} \frac{(y_j + z_{ij})(y_k + z_{ik})}{\|\boldsymbol{y} + \mathbf{z}_i\|_2^2} + \sum_{i=K'+1}^{K} \frac{z_{ij} z_{ik}}{\|\mathbf{z}_i\|_2^2} \\
&= \left(\sum_{i=1}^{K'} \|\boldsymbol{y} + \mathbf{z}_i\|_2^{-2} \right) y_j y_k + \left(\sum_{i=1}^{K'} z_{ik} \|\boldsymbol{y} + \mathbf{z}_i\|_2^{-2} \right) y_j + \left(\sum_{i=1}^{K'} z_{ij} \|\boldsymbol{y} + \mathbf{z}_i\|_2^{-2} \right) y_k + \sum_{i=1}^{K'} z_{ij} z_{ik} \|\boldsymbol{y} + \mathbf{z}_i\|_2^{-2} \\
&\quad + \sum_{i=K'+1}^{K} \frac{z_{ij} z_{ik}}{\|\mathbf{z}_i\|_2^2}
\end{aligned}$$

where $K' = (1 - \eta)K$. This implies that

$$\bar{\mathbf{X}}^\top \bar{\mathbf{X}} = \|\mathbf{g}\|_2^2 \cdot \boldsymbol{y}\boldsymbol{y}^\top + \boldsymbol{y}\mathbf{g}^\top \mathbf{H}^\top + \mathbf{H}\mathbf{g}\boldsymbol{y}^\top + \mathbf{H}\mathbf{H}^\top + \mathbf{L}\mathbf{L}^\top, \tag{B.44}$$

where

$$\mathbf{g} = (\|\boldsymbol{y} + \mathbf{z}_1\|_2^{-1}, \|\boldsymbol{y} + \mathbf{z}_2\|_2^{-1}, ..., \|\boldsymbol{y} + \mathbf{z}_{K'}\|_2^{-1}), \qquad \mathbf{H} = \left[\frac{\mathbf{z}_1}{\|\boldsymbol{y} + \mathbf{z}_1\|_2} \quad \frac{\mathbf{z}_2}{\|\boldsymbol{y} + \mathbf{z}_2\|_2} \quad \cdots \quad \frac{\mathbf{z}_{K'}}{\|\boldsymbol{y} + \mathbf{z}_{K'}\|_2} \right] \in \mathbb{R}^{n \times K'},$$

and

$$\mathbf{L} = \left[\frac{\mathbf{z}_{K'+1}}{\|\mathbf{z}_{K'+1}\|_2} \quad \cdots \quad \frac{\mathbf{z}_K}{\|\mathbf{z}_K\|_2} \right] \in \mathbb{R}^{n \times \eta K},$$

In other words,

$$\bar{\mathbf{X}}^\top \bar{\mathbf{X}} = \|\mathbf{g}\|_2^2 \cdot \boldsymbol{y}\boldsymbol{y}^\top + \mathbf{L}\mathbf{L}^\top + \mathbf{E}, \qquad \mathbf{E} = \boldsymbol{y}\mathbf{g}^\top \mathbf{H}^\top + \mathbf{H}\mathbf{g}\boldsymbol{y}^\top + \mathbf{H}\mathbf{H}^\top, \tag{B.45}$$

Note that by definition

$$\mathbf{L}\mathbf{L}^\top \boldsymbol{y} = 0,$$

then we can treat the above decomposition as a low-rank matrix $\|\mathbf{g}\|_2^2 \cdot \boldsymbol{y}\boldsymbol{y}^\top + \mathbf{L}\mathbf{L}^\top$ plus a noise matrix $\mathbf{E}$. In this respect, the Davis-Kahan's perturbation theorem applies similarly as in the proof of Theorem 1, only with $\frac{\|\mathbf{E}\|}{\|\mathbf{g}\|_2^2 \|\boldsymbol{y}\|_2^2}$ therein replaced by

$$\frac{\|\mathbf{E}\|}{\|\mathbf{g}\|_2^2 \|\boldsymbol{y}\|_2^2 - \|\mathbf{L}\|^2}.$$

Note that by definition we have

$$\|\mathbf{L}\| \leq \sqrt{\eta K}, \tag{B.46}$$

whereas by (B.23) we have

$$\|\mathbf{g}\|_2^2\|\boldsymbol{y}\|_2^2 \geq \frac{(1-\eta)K\|\boldsymbol{y}\|_2^2}{(1+\delta_n')(\|\boldsymbol{y}\|_2^2+c\sigma^2 n)}. \tag{B.47}$$

Then, as long as

$$\frac{1-\eta}{(1+\delta_n')(1+c\sigma^2 n/\|\boldsymbol{y}\|_2^2)} > 2\eta, \tag{B.48}$$

we have

$$\frac{\|\mathbf{E}\|}{\|\mathbf{g}\|_2^2\|\boldsymbol{y}\|_2^2-\|\mathbf{L}\|^2} \leq \frac{\|\mathbf{E}\|}{\eta K},$$

and the rest argument leading to (B.43) follows as in the proof of Theorem 2. Finally, note that a sufficient condition for (B.48) is

$$\|\boldsymbol{y}\|_2/\sigma \geq C\sqrt{n}, \qquad \eta < C^{-1}, \tag{B.49}$$

for some sufficiently large $C>0$. This completes the first part of the proof.

As for the second statement concerning $\bar{\mathbf{P}}^a$, note that by (B.25), whenever $\|\boldsymbol{y}\|_2/\sigma \geq C\sqrt{n}$ for some sufficiently large C, all the $(1-\eta)K$ candidate visualizations are concordant with the true structure in probability, that is $\cos\angle(\mathbf{x}_k,\boldsymbol{y}) \to_P 1$ for all $k\in\mathcal{C}_0$. In particular, we have

$$\sum_{k\in\mathcal{C}_0}[1-\cos\angle(\mathbf{x}_k,\boldsymbol{y})] \leq (1-\eta)K\epsilon/C,$$

in probability for some constant $\epsilon>0$. Without loss of generality, we assume $\boldsymbol{y}=(1,0,...0)$, and $\bar{\mathbf{x}}_k=(0,1,0,...,0)$ for all $k\in\mathcal{C}_1$. Thus, it follows that

$$\sum_{k=1}^{K}\bar{\mathbf{x}}_k = (0,\eta K,0,...,0)+((1-\eta)K,0,...,0)+\sum_{k=1}^{(1-\eta)K}(\bar{\mathbf{x}}_k-\boldsymbol{y}),$$

and

$$\begin{aligned}\|\sum_{k=1}^{K}\bar{\mathbf{x}}_k\|_2 &\geq \sqrt{\eta K^2+(1-\eta)^2K^2}-\sum_{k=1}^{(1-\eta)K}\|\bar{\mathbf{x}}_k-\boldsymbol{y}\|_2\\ &\geq K\sqrt{(\eta^2+(1-\eta)^2)}-2(1-\epsilon)K\epsilon/C,\end{aligned}$$

in probability. If C is chosen such that $\epsilon/C<0.01$, then we have

$$\cos\angle(\sum_{k=1}^{K}\bar{\mathbf{x}}_k,\boldsymbol{y}) \leq \frac{(1-\eta)K+2(1-\epsilon)K\epsilon/C}{K\sqrt{(\eta^2+(1-\eta)^2)}-2(1-\epsilon)K\epsilon/C} \leq \frac{1-\eta+0.02(1-\eta)}{\sqrt{1-2\eta+2\eta^2-0.02(1-\eta)}} < 1-\eta,$$

in probability.

B.6 Necessity of the Signal Strength Condition (C2)

In this part, we show that the signal strength condition (C2) required by the proposed method is in fact asymptotically sharp and optimal, in the sense that it is essentially required for any possible method in order to achieve consistent estimation of the true structure $\mathbf{P}^*$.

Specifically, we consider the settings where the following two conditions hold:
(C1c) $\{\mathbf{h}_i^{(k)}\}_{1\le k\le K}$ are identically distributed Gaussian random vectors with mean zero and covariance matrix $\sigma^2\mathbf{I}_n$.
(C1d) The matrix $\mathbf{R}=(\rho_{jk})_{1\le j,k\le K}$ satisfies $\rho:=\|\mathbf{R}\|=O(1)$.
Note that Conditions (C1c) and (C1d) are sufficient conditions for Conditions (C1a) and (C1b), respectively, so that Conditions (C1a) and (C1b) are satisfied throughout our discussion below.

Using the previous notations, we can rewrite our model for given i as

$$\mathbf{x}_k = c_k(\boldsymbol{y}+\mathbf{z}_k), \qquad k=1,2,...,K,$$

where by (C1c) $\{\mathbf{z}_k\}_{1\le k\le K}$ are identically distributed Gaussian vectors with mean zero and covariance matrix $\sigma^2\mathbf{I}_n$, and $\mathbf{R}\in\mathbb{R}^{K\times K}$ is defined similarly as in the main paper describing the correlation structure among $\{\mathbf{z}_k\}_{1\le k\le K}$. We consider the parameter space for the model parameters $(\{c_k\}_{1\le k\le K},\boldsymbol{y},\sigma,\mathbf{R})$ as

$$\Theta(t,\sigma,K,n)=\left\{\begin{matrix}\{c_k\}_{1\le k\le K}\subset\mathbb{R},\boldsymbol{y}\in\mathbb{R}^n,\|\boldsymbol{y}\|_2=t,\\ \sigma>0,\mathbf{R}\in\mathbb{R}^{K\times K},\|\mathbf{R}\|\le C\end{matrix}\right\},$$

for some sufficiently large universal constant $C>1$. Define a subset of $\Theta(t,\sigma,K,n)$ as

$$\Theta_0(t,\sigma,K,n)=\left\{c_k=1,\forall 1\le k\le K\boldsymbol{y}\in\mathbb{R}^n,\sigma>0,\mathbf{R}=\mathbf{I}_K\right\}.$$

From our previous analysis (Theorem 2), it follows that as long as $t/\sigma\gg\sqrt{n/K}$, the proposed method satisfies

$$\lim_{n\to\infty}\sup_{\theta\in\Theta(t,\sigma,K,n)}P_\theta(\cos\angle(\mathbf{x}^m,\boldsymbol{y})>1-\epsilon)=1, \tag{B.50}$$

for all $\epsilon>0$. Now we show the following minimax lower bound result, which shows that consistent estimation of $\boldsymbol{y}$ is never possible over the parameter space $\Theta(t,\sigma,K,n)$ for any estimator $\widehat{\boldsymbol{y}}$ whenever $t/\sigma\le C_1\sqrt{n/K}$ for some large constant $C_1>0$.

Theorem 4. *Under Conditions (C1c) and (C1d), there exists some absolute constant $C_1>0$ such that, as long as $t/\sigma\le C_1\sqrt{n/K}$, we have*

$$\lim_{n\to\infty}\inf_{\widehat{\boldsymbol{y}}}\sup_{\theta\in\Theta(t,\sigma,K,n)}P_\theta(\cos\angle(\widehat{\boldsymbol{y}},\boldsymbol{y})<0.7)>c_1, \tag{B.51}$$

for some absolute constant $c_1>0$.

Proof. To see this, note that

$$\inf_{\widehat{\boldsymbol{y}}}\sup_{\theta\in\Theta(t,\sigma,K,n)}P_\theta(\cos\angle(\widehat{\boldsymbol{y}},\boldsymbol{y})<0.7)\ge\inf_{\widehat{\boldsymbol{y}}}\sup_{\theta\in\Theta_0(t,\sigma,K,n)}P_\theta(\cos\angle(\widehat{\boldsymbol{y}},\boldsymbol{y})<0.7). \tag{B.52}$$

Over the parameter space $\Theta_0(t,\sigma,K,n)$, the model reduces to

$$\mathbf{X}=\mathbf{1}\boldsymbol{y}^\top+\mathbf{Z}\in\mathbb{R}^{K\times n}, \tag{B.53}$$

which is rank-one matrix denoising model, where $\|\mathbf{1}\boldsymbol{y}^\top\| = t\sqrt{K}$ and $\mathbf{Z}$ has *i.i.d.* rows drawn from $N(0, \sigma^2\mathbf{I}_n)$. The estimation of the direction of $\boldsymbol{y}$ is thus equivalent to estimation of the right singular vector of the rank-one matrix $\mathbf{1}\boldsymbol{y}^\top$. Now we recall the following lemma concerning the minimax lower bound for singular vector estimation in the low-rank matrix denoising model, proved by [5].

Lemma 2. *Let $\bar{\boldsymbol{y}} = \boldsymbol{y}/\|\boldsymbol{y}\|_2$. There exists some constants $c_1, c_2 > 0$ such that*

$$\inf_{\widehat{\boldsymbol{y}}} \sup_{\theta\in\Theta(t,\sigma,K,n)} P_\theta\left(\|\bar{\boldsymbol{y}}\bar{\boldsymbol{y}}^\top - \widehat{\boldsymbol{y}}\widehat{\boldsymbol{y}}^\top\|_F \geq \min\left\{1, \frac{c_1\sigma\sqrt{n}}{t\sqrt{K}}\right\}\right) \geq c_2, \tag{B.54}$$

for sufficiently large n.

As a result, since by Lemma 1 of [6], we have

$$\|\bar{\boldsymbol{y}}\bar{\boldsymbol{y}}^\top - \widehat{\boldsymbol{y}}\widehat{\boldsymbol{y}}^\top\|_F = \sqrt{2}\sin\angle(\boldsymbol{y}, \widehat{\boldsymbol{y}}) = \sqrt{1-\cos^2\angle(\boldsymbol{y}, \widehat{\boldsymbol{y}})} \leq \sqrt{2(1-\cos\angle(\boldsymbol{y}, \widehat{\boldsymbol{y}}))}. \tag{B.55}$$

Then, it follows that $\|\bar{\boldsymbol{y}}\bar{\boldsymbol{y}}^\top - \widehat{\boldsymbol{y}}\widehat{\boldsymbol{y}}^\top\|_F \geq \sqrt{0.6}$ implies $\cos\angle(\boldsymbol{y}, \widehat{\boldsymbol{y}}) < 0.7$. Therefore, whenever we choose a constant $C_1 > 0$ sufficiently large so that $\frac{c_1C_1\sigma\sqrt{n}}{t\sqrt{K}} > \sqrt{0.6}$, we have

$$\inf_{\widehat{\boldsymbol{y}}} \sup_{\theta\in\Theta(t,\sigma,K,n)} P_\theta(\|\bar{\boldsymbol{y}}\bar{\boldsymbol{y}}^\top - \widehat{\boldsymbol{y}}\widehat{\boldsymbol{y}}^\top\|_F \geq \sqrt{0.6}) \geq c_2, \tag{B.56}$$

which implies

$$\inf_{\widehat{\boldsymbol{y}}} \sup_{\theta\in\Theta(t,\sigma,K,n)} P_\theta(\cos\angle(\boldsymbol{y}, \widehat{\boldsymbol{y}}) < 0.7) \geq c_2, \tag{B.57}$$

This completes the proof of the theorem. □

B.7 Proof of Auxiliary Lemma 1

We need the following lemma, proved in [6], to obtain the upper bound.

Lemma 3. *For any $p \geq 1$, denote $\mathbb{B}^p = \{x \in \mathbb{R}^p : \|x\|_2 \leq 1\}$ as the p-dimensional unit ball in the Euclidean space. Suppose $K \in \mathbb{R}^{p_1\times p_2}$ is a random matrix. Then we have for $t > 0$,*

$$P(\|K\| \geq 3t) \leq 7^{p_1+p_2} \cdot \max_{u\in\mathbb{B}^{p_1}, v\in\mathbb{B}^{p_2}} P(|u^\top Kv| \geq t).$$

By Lemma 3, for $\mathbf{H}_0$, it follows that

$$P(\|\mathbf{H}_0\| \geq 3t) \leq 7^{n+d} \cdot \max_{\mathbf{u}\in\mathbb{B}_n(1), \mathbf{v}\in\mathbb{B}_d(1)} P(|\mathbf{u}^\top\mathbf{H}_0\mathbf{v}| \geq t). \tag{B.58}$$

For any $\mathbf{u} \in \mathbb{B}_n(1)$ and $\mathbf{v} \in \mathbb{B}_d(1)$, we have

$$\mathbf{u}^\top\mathbf{H}_0\mathbf{v} = \mathrm{tr}(\mathbf{H}_0\mathbf{v}\mathbf{u}^\top) = \langle\mathbf{H}_0, \mathbf{u}\mathbf{v}^\top\rangle, \tag{B.59}$$

where $\langle\cdot,\cdot\rangle$ is Hilbert-Schmidt norm for matrices. Now define $\boldsymbol{\Sigma} \in \mathbb{R}^{nd\times nd}$ be $\mathbb{E}[\mathrm{vec}(\mathbf{H}_0)\mathrm{vec}(\mathbf{H}_0)^\top]$, so that its (i,j)-th block is $\boldsymbol{\Sigma}_{ij}$. By Conditions (C1a) and (C1b) and the definition of $\{\rho_{ij}\}$, we have

$$\boldsymbol{\Sigma} = \begin{bmatrix} \boldsymbol{\Sigma}_{11} & \boldsymbol{\Sigma}_{12} & ... & \boldsymbol{\Sigma}_{1d} \\ \boldsymbol{\Sigma}_{21} & \boldsymbol{\Sigma}_{22} & .. & \boldsymbol{\Sigma}_{2d} \\ \vdots & & & \\ \boldsymbol{\Sigma}_{d1} & \boldsymbol{\Sigma}_{d2} & .. & \boldsymbol{\Sigma}_{dd} \end{bmatrix}$$

where $\|\mathbf{\Sigma}_{ij}\| = \sigma^2\rho_{ij}$ for $1 \le i, j \le d$. In particular, we have

$$\|\mathbf{\Sigma}\| \le \sigma^2\|\mathbf{R}\| = \sigma^2\rho. \tag{B.60}$$

To see this, if we denote $\mathbf{v} = (\mathbf{v}_1, ..., \mathbf{v}_d)^\top \in \mathbb{R}^{nd}$, then

$$\begin{aligned}\|\mathbf{\Sigma}\| &= \max_{\|\mathbf{v}\|_2=1} \mathbf{v}^\top\mathbf{\Sigma}\mathbf{v} = \max_{\|\mathbf{v}\|_2=1} \sum_{1\le i,j\le d} \mathbf{v}_i^\top\mathbf{\Sigma}_{ij}\mathbf{v}_j \le \max_{\|\mathbf{v}\|_2=1} \sum_{1\le i,j\le d} \|\mathbf{v}_i\|_2\|\mathbf{v}_j\|_2\|\mathbf{\Sigma}_{ij}\| \\ &= \sigma^2 \max_{\|\mathbf{l}\|_2=1} \mathbf{l}^\top\mathbf{R}\mathbf{l} = \|\mathbf{R}\|.\end{aligned}$$

Now by (B.60), we have

$$\mathbb{E}\langle\mathbf{H}_0, \mathbf{u}\mathbf{v}^\top\rangle \le C\sigma\sqrt{\rho}. \tag{B.61}$$

By properties of sub-Gaussian vectors, it holds that

$$P(|\langle\mathbf{H}_0, \mathbf{u}\mathbf{v}^\top\rangle| \le C\sigma\sqrt{\rho}(1+t)) \ge 1 - e^{-ct^2}. \tag{B.62}$$

Combining (B.62) and (B.58), we have

$$\begin{aligned}P(\|\mathbf{H}_0\| \ge 3C\sigma\sqrt{\rho}(1+t)) &\le 7^{n+d}P(|\mathbf{u}^\top\mathbf{H}_0\mathbf{v}| \ge C\sigma\sqrt{\rho}(1+t)) \\ &\le 7^{n+d}e^{-ct^2} \\ &\le \exp\{c'(n+d) - ct^2\}.\end{aligned}$$

Setting $t = c_1(\sqrt{n} + \sqrt{d})$ for some sufficiently large $c_1 > 0$, we have

$$P(\|\mathbf{H}_0\| \ge C\sigma\sqrt{\rho}(\sqrt{n} + \sqrt{d})) \le n^{-c}. \tag{B.63}$$

Supplementary References

[1] Matthew T Buckley, Eric Sun, Benson M George, Ling Liu, Nicholas Schaum, Lucy Xu, Jaime M Reyes, Margaret A Goodell, Irving L Weissman, Tony Wyss-Coray, et al. Cell type-specific aging clocks to quantify aging and rejuvenation in regenerative regions of the brain. *bioRxiv*, 2022.

[2] Roman Vershynin. *High-dimensional probability: An introduction with applications in data science*, volume 47. Cambridge University Press, 2018.

[3] Rajendra Bhatia. *Matrix Analysis*, volume 169. Springer Science & Business Media, 2013.

[4] Yi Yu, Tengyao Wang, and Richard J Samworth. A useful variant of the davis–kahan theorem for statisticians. *Biometrika*, 102(2):315–323, 2015.

[5] T Tony Cai, Hongzhe Li, and Rong Ma. Optimal structured principal subspace estimation: Metric entropy and minimax rates. *J. Mach. Learn. Res.*, 22:46–1, 2021.

[6] T Tony Cai and Anru Zhang. Rate-optimal perturbation bounds for singular subspaces with applications to high-dimensional statistics. *The Annals of Statistics*, 46(1):60–89, 2018.
