# Supplementary material for: A spectral method for assessing and combining multiple data visualizations
Source: Nat Commun. 2023 Feb 11;14:780. doi: 10.1038/s41467-023-36492-2 (PMC9922271; doi:10.1038/s41467-023-36492-2)
Supplement: Supplementary file 3 — Reporting Summary [file 41467_2023_36492_MOESM3_ESM.pdf]

nature portfolio

Corresponding author(s): James Zou

Last updated by author(s): Jan 19, 2023

Reporting Summary

Nature Portfolio wishes to improve the reproducibility of the work that we publish. This form provides structure for consistency and transparency in reporting. For further information on Nature Portfolio policies, see our Editorial Policies and the Editorial Policy Checklist.

Statistics

For all statistical analyses, confirm that the following items are present in the figure legend, table legend, main text, or Methods section.

n/a	Confirmed	
☐	☒	The exact sample size (*n*) for each experimental group/condition, given as a discrete number and unit of measurement
☐	☒	A statement on whether measurements were taken from distinct samples or whether the same sample was measured repeatedly
☐	☒	The statistical test(s) used AND whether they are one- or two-sided<br>*Only common tests should be described solely by name; describe more complex techniques in the Methods section.*
☐	☒	A description of all covariates tested
☐	☒	A description of any assumptions or corrections, such as tests of normality and adjustment for multiple comparisons
☐	☒	A full description of the statistical parameters including central tendency (e.g. means) or other basic estimates (e.g. regression coefficient) AND variation (e.g. standard deviation) or associated estimates of uncertainty (e.g. confidence intervals)
☒	☐	For null hypothesis testing, the test statistic (e.g. *F*, *t*, *r*) with confidence intervals, effect sizes, degrees of freedom and *P* value noted<br>*Give P values as exact values whenever suitable.*
☒	☐	For Bayesian analysis, information on the choice of priors and Markov chain Monte Carlo settings
☒	☐	For hierarchical and complex designs, identification of the appropriate level for tests and full reporting of outcomes
☐	☒	Estimates of effect sizes (e.g. Cohen's *d*, Pearson's *r*), indicating how they were calculated

Our web collection on statistics for biologists contains articles on many of the points above.

Software and code

Policy information about availability of computer code

Data collection	Not applicable.
Data analysis	Data analysis code is available at https://github.com/rongstat/meta-visualization. We used the following versions of R packages across all analyses: rARPACK (0.11-0), MASS (7.3-58.1), lle (1.1), dimRed (0.2.6), uwot (0.1.14), cluster (2.1.4), Rtsne (0.16), phateR (1.0.7), Seurat (4.3.0), ggplot2 (3.4.0), ggrepel (0.9.1), data.table (1.14.6), RColorBrewer (1.1-3), dplyr (1.0.10), Rfast (2.0.6).

For manuscripts utilizing custom algorithms or software that are central to the research but not yet described in published literature, software must be made available to editors and reviewers. We strongly encourage code deposition in a community repository (e.g. GitHub). See the Nature Portfolio guidelines for submitting code & software for further information.

Data

Policy information about availability of data

All manuscripts must include a data availability statement. This statement should provide the following information, where applicable:

- Accession codes, unique identifiers, or web links for publicly available datasets
- A description of any restrictions on data availability
- For clinical datasets or third party data, please ensure that the statement adheres to our policy

The religious and biblical text data are downloaded from UCI Machine Learning Repository [https://archive.ics.uci.edu/ml/machine-learning-databases/00512/]. The cell cycle analysis is based on the mouse embryonic stem cell data, available in EMBL-EBI with accession code E-MTAB-2805 [https://www.ebi.ac.uk/biostudies/

March 2021

arrayexpress/studies/E-MTAB-2805]. The cell trajectory analysis is based on the mouse embryonic stem single-cell data, available in Gene Expression Omnibus with accession code GSE98664 [https://www.ncbi.nlm.nih.gov/geo/query/acc.cgi?acc=GSE98664]. The single-cell transcriptomic dataset used for evaluating computational cost is accessible at BioProject with accession code PRJNA795276 [https://www.ncbi.nlm.nih.gov/bioproject/?term=PRJNA297956].

Human research participants

Policy information about studies involving human research participants and Sex and Gender in Research.

Reporting on sex and gender	The datasets analyzed in this paper do not contain sex information.
Population characteristics	N/A
Recruitment	N/A
Ethics oversight	N/A

Note that full information on the approval of the study protocol must also be provided in the manuscript.

Field-specific reporting

Please select the one below that is the best fit for your research. If you are not sure, read the appropriate sections before making your selection.

☒ Life sciences ☐ Behavioural & social sciences ☐ Ecological, evolutionary & environmental sciences

For a reference copy of the document with all sections, see nature.com/documents/nr-reporting-summary-flat.pdf

Life sciences study design

All studies must disclose on these points even when the disclosure is negative.

Sample size	Not applicable. We used publicly available datasets.
Data exclusions	Our analysis of the religious text data, the cell cycle data, and the cell trajectory data did not exclude samples from the original datasets. In the analysis of the mouse neurogenic region single-cell transcriptomic dataset, we randomly subsampled the data in order to create datasets of different sample sizes to evaluate computational time.
Replication	Random seeds were set for algorithms that involve randomized steps. The R codes used for generating all the numerical results are available at our GitHub repository https://github.com/rongstat/meta-visualization.
Randomization	Not applicable. This study only used publicly available datasets and the randomization for those studies can be found in their original publications.
Blinding	Not applicable. This study only used publicly available datasets and blinding procedures for individual datasets can be found in their original publications. The investigators were not blinded to the datasets used in the experiments presented in the manuscript.

Reporting for specific materials, systems and methods

We require information from authors about some types of materials, experimental systems and methods used in many studies. Here, indicate whether each material, system or method listed is relevant to your study. If you are not sure if a list item applies to your research, read the appropriate section before selecting a response.

Materials & experimental systems

n/a	Involved in the study	
☒	☐	Antibodies
☒	☐	Eukaryotic cell lines
☒	☐	Palaeontology and archaeology
☒	☐	Animals and other organisms
☒	☐	Clinical data
☒	☐	Dual use research of concern

Methods

n/a	Involved in the study	
☒	☐	ChIP-seq
☒	☐	Flow cytometry
☒	☐	MRI-based neuroimaging
